# One-pot synthesis of hetero[6]rotaxane bearing three different kinds of macrocycle through a self-sorting process†
†Electronic supplementary information (ESI) available. See DOI: 10.1039/c7sc03232c



**DOI:** 10.1039/c7sc03232c

**Published:** 2017-08-04

**Authors:** Si-Jia Rao, Qi Zhang, Ju Mei, Xu-Hao Ye, Chuan Gao, Qiao-Chun Wang, Da-Hui Qu, He Tian

**Affiliations:** a Key Laboratory for Advanced Materials , Institute of Fine Chemicals , School of Chemistry and Molecular Engineering , East China University of Science and Technology , 130 Meilong Road , Shanghai , 200237 , China . Email: dahui_qu@ecust.edu.cn

## Abstract

In this article, a six-component self-sorting process that involves three types of crown ether macrocycle and three types of cation guest molecule was carefully and thoroughly investigated. The six components include three kinds of crown ether, namely bis(*p*-phenylene-34-crown-10) (BPP34C10), dibenzo-24-crown-8 (DB24C8) and benzo-21-crown-7 (B21C7), and their corresponding cation guest molecules, namely a 4,4′-bipyridine dication (BPY^2+^) and dibenzylammonium (DBA) and benzylalkylammonium (BAA) ions, respectively. Based on this well-established highly selective six-component self-sorting process, a hetero[6]rotaxane bearing three different kinds of crown ether macrocycle was designed and successfully synthesized through a facile and efficient one-pot “click” stoppering strategy. Such work is proposed to be a significant advance in the construction of mechanically interlocked molecules with high structural complexity, as well as a good supplement in the areas of multi-component self-sorting and noncovalent self-assembly.

## Abstract 2

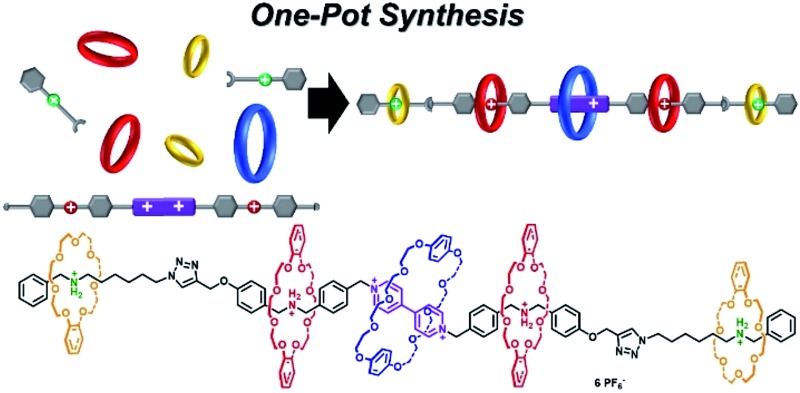
A hetero[6]rotaxane bearing three different kinds of macrocycle is designed and successfully synthesized through a one-pot “click” reaction by employing a facile and efficient integrative self-sorting principle.

## Introduction

In naturally occurring systems, the principles of reversible noncovalent self-assembly and self-sorting are widely used to construct complex architectures from several different building blocks to execute important tasks and specific functions.^1^ Inspired by nature, a variety of complex and well-ordered multicomponent molecular or supramolecular systems have been constructed or assembled *via* noncovalent synthetic strategies.^2^ Rotaxanes,^3,4^ well-known for their unique structures consisting of mechanically interlocked threads and macrocycles, have attracted considerable attention in the past few decades due to their remarkable potential to evolve into molecular switches and molecular machines.^5^ Towards achieving multistate and multifunctional rotaxane-based molecular systems, chemists have been putting unremitting effort into the pursuit of increasing the structural complexity of mechanically interlocked molecules^6^ and other chemical topological structures.^7^ Owing to the diversity of macrocycles and their host-guest systems, various rotaxanes bearing one or more types of macrocycle, also called hetero[*n*]rotaxanes,^8^ have been successfully designed and constructed.

There have been some elegant examples of hetero[*n*]rotaxanes containing two different macrocycles,^9^ which were constructed using efficient self-sorting and an orthogonal self-assembly synthetic approach. Schalley^6*a*^ and co-workers reported the self-sorting process of two classes of macrocyclic polyether, DB24C8 and B21C7, and two sorts of ammonium cation, dibenzylammonium (DBA) and benzylalkylammonium (BAA) ions, and then used the concept of integrative self-sorting to prepare a hetero[3]rotaxane. By precisely programming the association constants and steric hindrance,^6a,b,9b,c^ several hetero[*n*]rotaxanes with high structural complexity, for example, Liu’s^9*a*^ twin-axle hetero[7]rotaxane, can be efficiently constructed by a facile one-pot route. Moreover, Stoddart^8a–c^ recently presented an effective “cooperative capture” strategy to construct a series of heterorotaxanes with two different kinds of macrocyclic ring, such as cucurbiturils and cyclodextrins or cucurbiturils and pillarenes, in good yields and high stereospecificities. Goldup^9*d*^ and co-workers introduced a proof-of-concept kinetic self-sorting approach for the efficient preparation of heterocircuit [3]rotaxanes. However, successful examples of heterorotaxanes bearing three or more different macrocycles have rarely been reported,^9*e*^ which would provide more possibilities for functionalization^10^ towards more advanced and complex molecular systems. The major difficulty lies in the remarkably increased number of possible assembly modes in the presence of different kinds of macrocycle and guest molecule in a single system. The design of an ultimate specific and selective self-sorting system,^11^ which can be introduced as a powerful tool for the efficient construction of complex hetero[*n*]rotaxanes bearing different kinds of macrocycle, is of great importance and highly desirable.

**Scheme 1:**
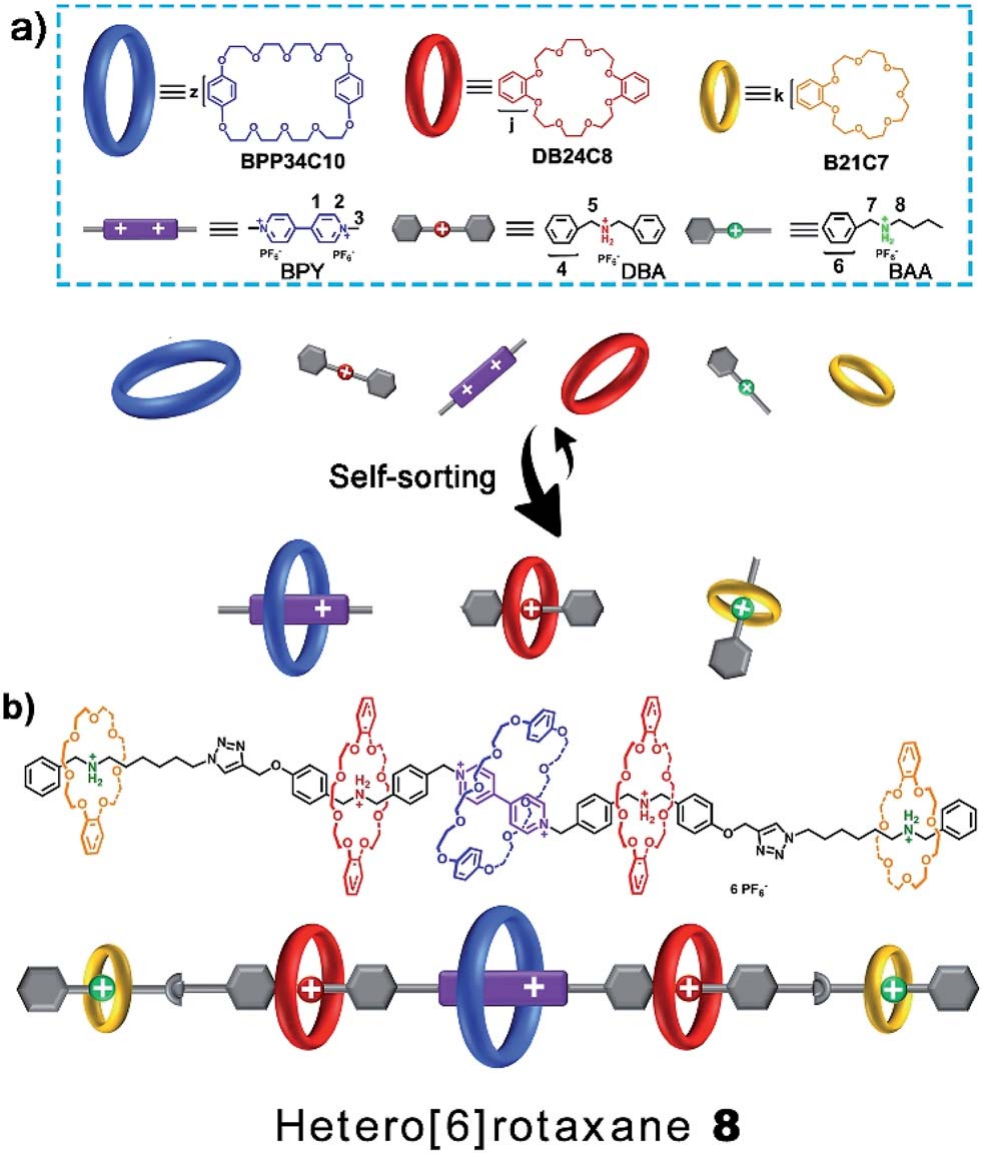
(a) Schematic representation of the six-component self-sorting process to predominantly form three interlocked species: BPP34C10 ⊃ BPY^2+^, DB24C8 ⊃ DBA and B21C7 ⊃ BAA. (b) Molecular structure and cartoon picture of the hetero[6]rotaxane **8** which has three different crown ether macrocycles, namely BPP34C10, DB24C8 and B21C7.

In this article, we present herein an efficient six-component integrative self-sorting process among three types of crown ether macrocycle and three types of guest molecule. Based on this well-established six-component integrative self-sorting process with good selectivity, a hetero[6]rotaxane bearing three different kinds of crown ether macrocycle was designed and successfully synthesized through a facile and efficient one-pot threading-followed-by-stoppering strategy (Scheme 1). We envisage that this work will present an important advance in the design and construction of complicated mechanically interlocked structures.

## Results and discussion

Macrocyclic polyethers,^12^ for example BPP34C10, DB24C8 and B21C7, have been widely employed in the fabrication of mechanically interlocked molecules and supramolecular polymers due to their host–guest interactions with versatile threadlike guests, such as, 4,4′-bipyridine dications (BPY^2+^) and secondary DBA and BAA salts, respectively. BPP34C10, a larger macrocycle than DB24C8, could recognize viologen units by strong charge transfer and π–π stacking interactions (*K*_a1_ = 240 M^–1^ in CH_3_CN),^12*e*^ while the affinity constant between DB24C8 and a viologen unit is *K*_a2_ = 31 M^–1^ in CH_3_CN,^12*f*^ which is much smaller than that between BPP34C10 and a viologen unit. Meanwhile, the hydrogen bonding between DB24C8 and a DBA site (*K*_a3_ = 420 M^–1^ in CH_3_CN)^12*g*^ was also proven to be stronger than that between BPP34C10 and a DBA site, and the binding constant for BPP34C10 and DBA is much weaker,^12*h*^ thus the elegant four-component self-sorting process between two macrocyclic rings (BPP34C10 and DB24C8) and two guest molecules (viologen and DBA) has been utilized in the orthogonal supramolecular polymerization of the two species of macrocycles and the two corresponding species of guest molecules.^13^ Moreover, the other four-component self-sorting process between DB24C8, B21C7, and DBA and BAA units has been demonstrated.^6*a*^ Hence, herein our motivation is to integrate the above-mentioned two kinds of four-component self-sorting system into an unprecedented six-component self-sorting system based on macrocyclic polyethers, including three types of macrocycle, BPP34C10, DB24C8 and B21C7, and their three corresponding guest species, viologen, DBA and BAA units, then to utilize this unique self-sorting strategy to construct an unprecedented [6]rotaxane with three distinct macrocycles (Scheme 1).

**Fig. 1:**
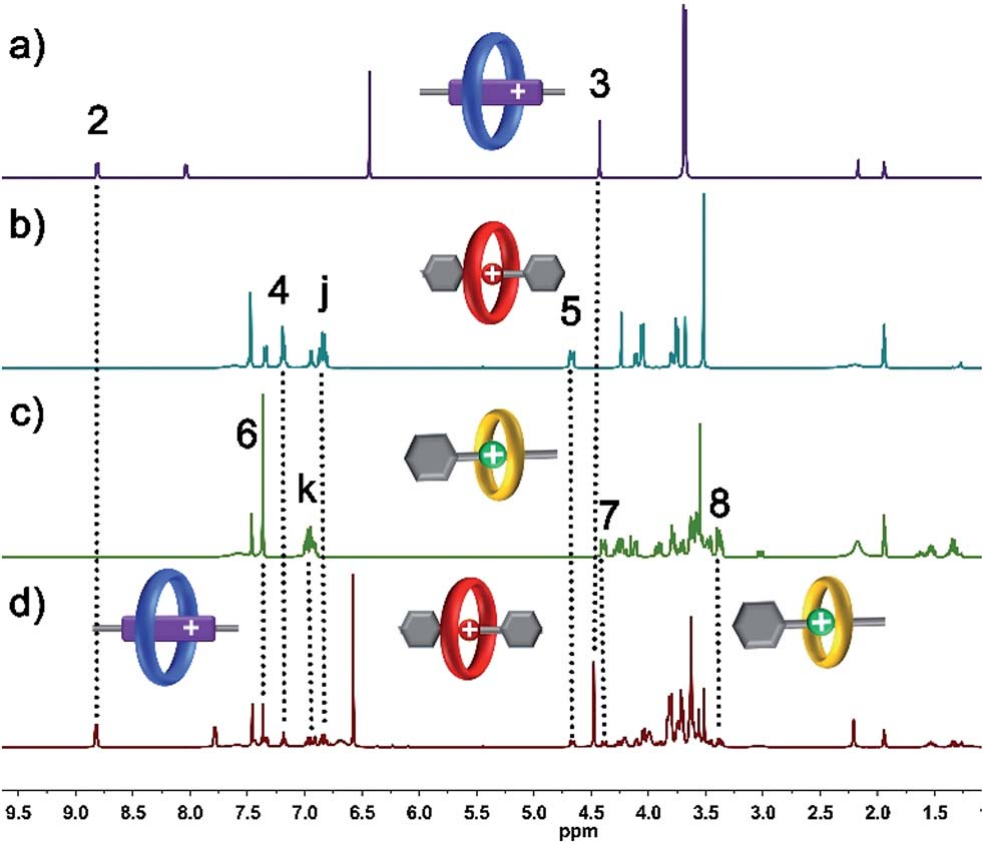
Partial ^1^H NMR spectra (400 MHz, 298 K, CD_3_CN) of (a) 1 : 1 mixture of BPP34C10 ⊃ BPY^2+^, (b) 1 : 1 mixture of DB24C8 ⊃ DBA, (c) 1 : 1 mixture of B21C7 ⊃ BAA and (d) equal molar mixture of BPP34C10, DB24C8, B21C7, BPY^2+^, DBA and BAA.

The six-component self-sorting process was thoroughly investigated through a comparison of ^1^H NMR spectra of the different combinations of crown ether rings and corresponding guest molecules (Fig. 1). Firstly, BPP34C10 and BPY^2+^ were mixed in CD_3_CN in the same molar ratio, and the ^1^H NMR spectrum (Fig. 1a and S1 in the ESI†) clearly confirmed the formation of the pseudorotaxane BPP34C10 ⊃ BPY^2+^ due to strong charge transfer and π–π stacking interactions. Similarly, the host–guest interactions between DB24C8 and DBA and B21C7 and BAA were also confirmed by the comparison of different ^1^H NMR spectra (see Fig. S2 and S3 in the ESI†), which fully demonstrate the formation of pseudorotaxanes DB24C8 ⊃ DBA and B21C7 ⊃ BAA (Fig. 1b and c) respectively. Next, we focused on the six-component self-sorting process. Fig. 1d shows the ^1^H NMR spectrum of the simple mixture of the three macrocyclic crown ether rings and the three corresponding guest molecules with different sizes and shapes. Interestingly, after careful analysis, we found that the ^1^H NMR spectrum (Fig. 1d) of an equal molar mixture of the six components showed the same pattern as the simple spectral overlap of Fig. 1a–c, evidenced by there being no obvious changes in the chemical shifts of the protons H_2_–H_8_, which indicated that the pseudorotaxanes BPP34C10 ⊃ BPY^2+^, DB24C8 ⊃ DBA and B21C7 ⊃ BAA are the predominant species in this multi-component self-assembly process. This unique self-sorting process could be attributed to the following encoded structural features: (i) BPP34C10 bears a larger binding constant with the BPY^2+^ site compared to that of DB24C8, hence BPP34C10 binds to the BPY^2+^ unit prior to DB24C8;^14^ (ii) DB24C8 enjoys the biggest binding constant with the DBA site compared to with the BAA or BPY^2+^ sites;^7*c*^ (iii) the binding behaviour between B21C7 and DBA is inhibited by the benzene rings of the DBA units,^6a,15^ leading to the specific combination with the guest BAA units. These factors synergistically resulted in a highly selective six-component self-sorting process, as shown in Scheme 1.

**Scheme 2:**
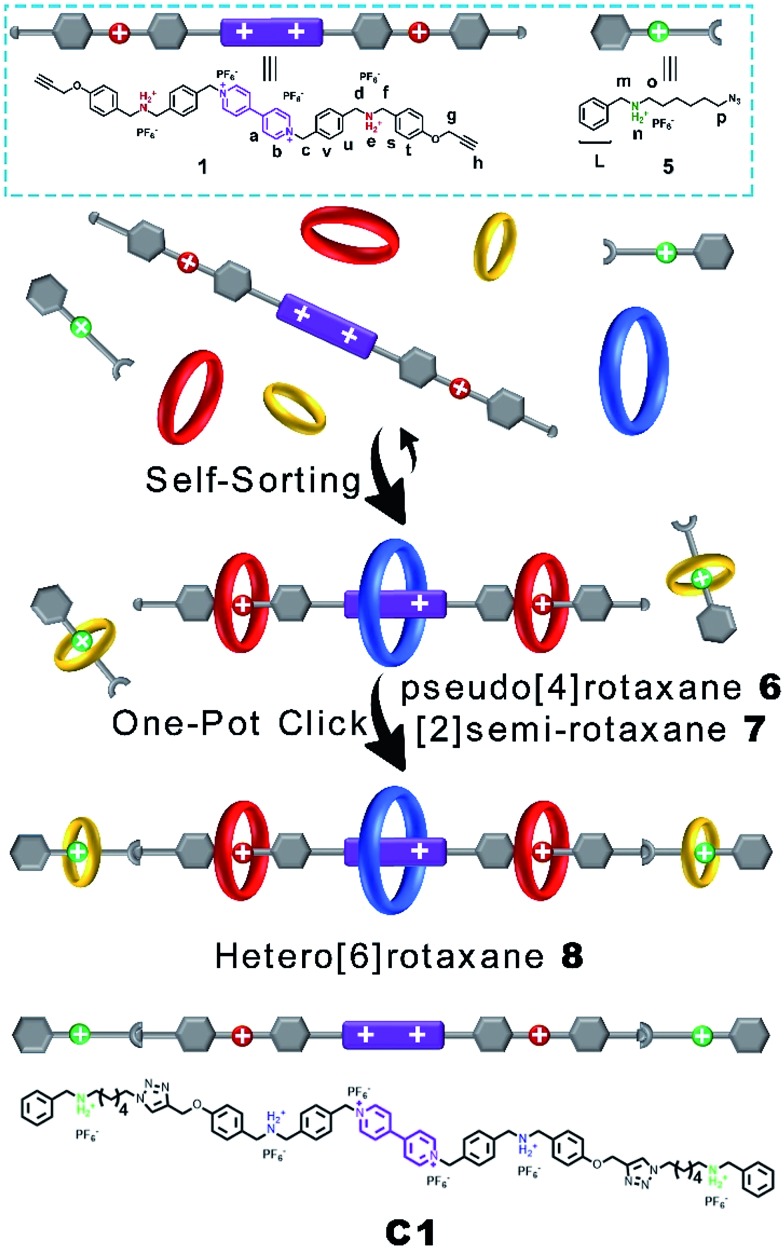
Schematic representation of the preparation of compound **C1** from **1** and **5** and hetero[6]rotaxane **8** from a five-component modularized self-sorting process of **1** and **5** and BPP34C10, DB24C8 and B21C7.

Then, we demonstrated for the first time the design and synthesis of a hetero[6]rotaxane bearing three different kinds of crown ether macrocycle *via* a facile and efficient one-pot strategy through a well-established self-sorting process among several types of host and guest species. As shown in Scheme 2, three macrocyclic polyethers with different cavity sizes, *i.e.* BPP34C10, DB24C8 and B21C7, were mixed with thread **1** and **5** according to the stoichiometric ratio in a single system. Although there were many possible assemblies among these precursors, only two types of assembly were formed in a specific and efficient self-sorting process, namely pseudo[4]rotaxane **6** consisting of BPP34C10, DB24C8 and thread **1**, and [2]semi-rotaxane **7** composed of B21C7 and thread **5**. Following the efficient self-sorting process which afforded pseudo[4]rotaxane **6** and [2]semi-rotaxane **7**, a cascade-stopped hetero[6]rotaxane **8** could be obtained by a facile one-pot copper(i)-catalyzed alkyne–azide cycloaddition (CuAAC) “click” reaction with a moderate yield of 49%.

**Fig. 2:**
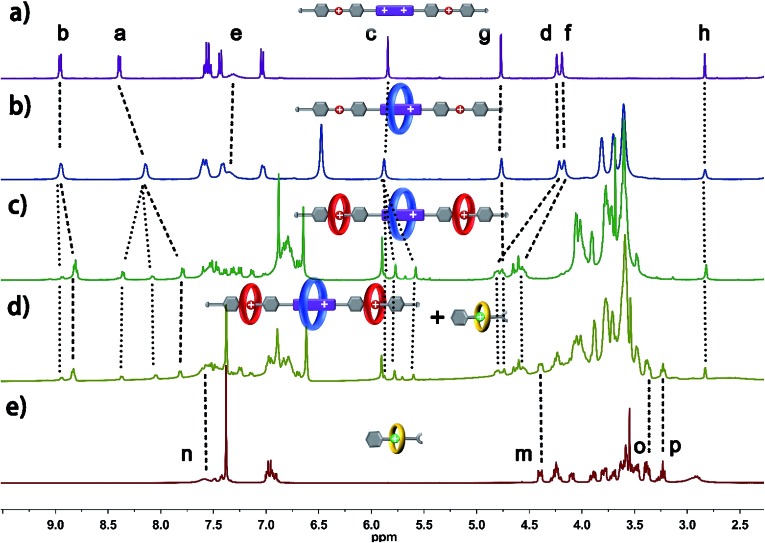
Partial ^1^H NMR spectra (400 MHz, CD_3_CN, 298 K) of (a) compound **1**, (b) 1 : 1 mixture of **1** and BPP34C10, (c) 1 : 1 : 2 mixture of **1**, BPP34C10 and DB24C8, (d) equal molar mixture of [4]pseudorotaxane **6** and [2]semi-rotaxane **7**, and (e) 1 : 1 mixture of **5** and B21C7.

The five-component self-sorting process which involves **1** and **5** and three kinds of crown ether was investigated using ^1^H NMR spectroscopy (Fig. 2). The ^1^H NMR spectrum of compound **1** in CD_3_CN was shown in Fig. 2a. Upon the addition of one molar equivalent of macrocycle BPP34C10, the strong host–guest interaction between BPP34C10 and the viologen unit on component **1** drove the efficient formation of the pseudo[2]rotaxane shown in Fig. 2b. Compared to the proton peaks exhibited in Fig. 2a, the aromatic protons H_a_ and H_b_ of the viologen unit in **1** were shifted upfield^16^ with Δ*δ* = 0.25 ppm for H_a_ and Δ*δ* = 0.05 ppm for H_b_, which was attributed to the deshielding effect exerted by the macrocycle BPP34C10. No obvious change was observed for the signal peaks of the H_g_, H_d_, H_f_ and H_h_ protons in the DBA moieties. All of these results indicate that the BPP34C10 ring rested on the viologen sites of **1** instead of on the DBA sites.

In the mixture of thread **1**, BPP34C10 and DB24C8 with a molar ratio of 1 : 1 : 2, pseudo[4]rotaxane **6** was formed, as evidenced by the ^1^H NMR spectrum in Fig. 2c. In contrast to that shown in Fig. 2b, the H_b_ proton signal split into two different peaks and one of them shifted upfield with a Δ*δ* value of 0.36 ppm. H_a_ split into three different peaks, one of which shifted upfield with a Δ*δ* value of 0.20 ppm, while the other two shifted downfield with Δ*δ* = 0.06 and 0.35 ppm respectively, due to the deshielding effect of the oxygen atoms on DB24C8 and the incomplete combination of DB24C8 and the dibenzylammonium unit.^14^ For the same reason, the resonance signal of H_c_ split into three different peaks. Since H_g_ and H_h_ did not directly interact with DB24C8, their chemical shifts were barely changed. H_d_ and H_f_ shifted downfield to Δ*δ* values of about 0.59 and 0.43 ppm, respectively. H_d_ and H_f_ also shifted due to the deshielding effect of the oxygen atoms from DB24C8.

On the other hand, the 1 : 1 mixture of B21C7 and component **5** in CD_3_CN could afford [2]semi-rotaxane **7**,^9*b*^ which was demonstrated by the downfield shift of the resonance peaks of H_m_ and H_p_ shown in Fig. 2e and S5 in the ESI.† It is worth mentioning that, upon mixing [2]semi-rotaxane **7** with pseudo[4]rotaxane **6** in a 1 : 1 molar ratio, no peak shift is observed in Fig. 2d, compared to either [2]semi-rotaxane **7** (Fig. 2e) or pseudo[4]rotaxane **6** (Fig. 2c). It should be noted that simultaneously mixing the five corresponding threads and macrocycles in one-pot at a molar ratio of 1 : 2 : 2 : 1 : 2 (BPP34C10 : DB24C8 : B21C7 : **1** : **5**) in CD_3_CN (Fig. S6†) gave the same ^1^H NMR spectral pattern as that in Fig. 2d. Such a result suggests the strong independence between these two types of assembly despite the dynamic nature of pseudo[4]rotaxane **6** and [2]semi-rotaxane **7**. These results indicated a highly selective self-sorting process among these five classes of component including three different kinds of macrocyclic polyether. This unique self-sorting process gave rise to the efficient and specific formation of pseudo[4]rotaxane **6** and [2]semi-rotaxane **7**, which could provide an effective pre-assembly template for the one-pot facile synthesis of target hetero[6]rotaxane **8** *via* a subsequent CuAAC stoppering reaction.

The structure of hetero[6]rotaxane **8** was confirmed by a variety of characterization tools, including ^1^H NMR, HRMS (Fig. S31–S35†), ^1^H–^1^H COSY (Fig. S9†) and ^1^H–^1^H NOESY (Fig. S10†). The major signal peaks in the HRMS spectrum of hetero[6]rotaxane **8** are found at *m*/*z* 1939.7592, 1244.8542, 897.3967, 688.9200, and 549.9410, which correspond to the target product after the loss of 2, 3, 4, 5 and 6 PF_6_^–^ ions, *i.e.* [M-2PF_6_]^2+^, [M-3PF_6_]^3+^, [M-4PF_6_]^4+^, [M-5PF_6_]^5+^ and [M-6PF_6_]^6+^, respectively. The ^1^H–^1^H NOESY spectrum of hetero[6]rotaxane **8** shows cross peaks (CP1 and CP2) between the protons of the BPP34C10 ring and the protons of the BPY^2+^ unit (H_b_ and H_a_). The cross peaks CP3 and CP5 suggest the correlation between the protons of the DB24C8 ring and the aromatic protons H_u_ and H_s_. The cross peak CP4 illustrates the relationship between the protons of the B21C7 ring and the aromatic protons H_L_. This means that we have successfully synthesized hetero[6]rotaxane *via* a self-sorting strategy using a one-pot mild CuAAC “click” reaction.

**Fig. 3:**
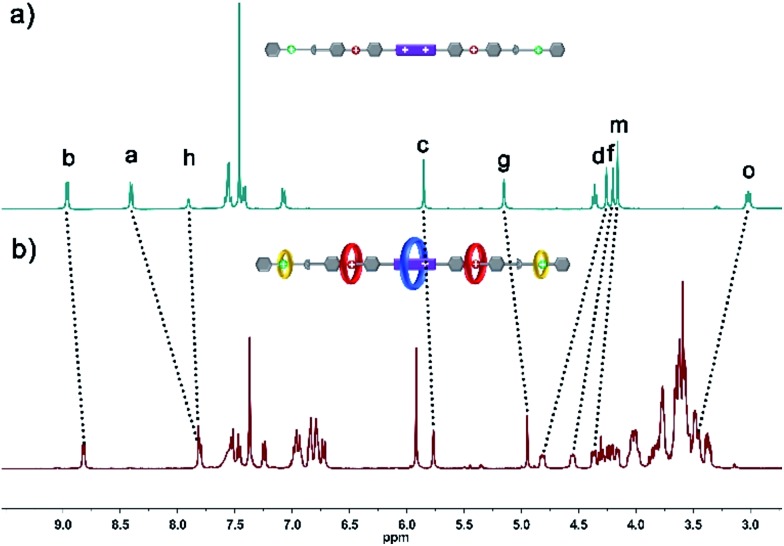
Partial ^1^H NMR spectra (400 MHz, 298 K, CD_3_CN) of (a) compound **C1** and (b) hetero[6]rotaxane **8**.

In order to gain deeper insight into the self-sorting process and further verification of the formation of the interlocked molecular hetero[6]rotaxane **8**, the axle compound **C1** (Scheme S1†) was also synthesized. Compounds **1**, **2** and Cu(CH_3_CN)_4_PF_6_ were mixed in CH_3_CN and stirred under the protection of argon at room temperature for 48 h, and the axle compound **C1** was obtained in a moderate yield of 37%. Compound **C1** was characterized by ^1^H NMR and ^13^C NMR spectroscopy and HRMS (Fig. S27–S30†). By comparing the ^1^H NMR spectra of **C1** and **8**, as shown in Fig. 3a and b, we can find the chemical shift changes of several peaks, which can be attributed to the fact that BPP34C10 and BPY^2+^, DB24C8 and DBA, and B21C7 and BAA could bind to each other *via* π–π stacking and hydrogen-bonding interactions. Owing to the deshielding effect of the combination of BPP34C10 and the viologen unit of BPY^2+^, the signals of H_b_ and H_a_ shifted upfield with Δ*δ* = –0.14 and –0.60 ppm, respectively. Meanwhile, due to the shielding effect of BPP34C10, the proton peaks of H_c_ shifted upfield with a Δ*δ* value of about –0.09 ppm. The signals of H_g_ shifted upfield with a Δ*δ* value of about –0.20 ppm, due to the shielding effect of the complexation of DB24C8 and the DBA unit. The signals of H_d_ and H_f_ shifted downfield with Δ*δ* = 0.56 and 0.35 ppm, respectively, owing to the deshielding effect of DB24C8. The signals of H_m_ and H_o_ were shifted downfield with Δ*δ* = 0.21 and Δ*δ* = 0.44 ppm, respectively, due to the shielding effect of the combination of B21C7 and the BAA unit. Hence the comparison of the ^1^H NMR spectra of **C1** and **8** provided further evidence for the confirmation of the proposed structure of hetero[6]rotaxane **8**.

## Experimental

### General methods

^1^H NMR spectra were recorded at 298 K using a Brüker AV-400 spectrometer at a frequency of 400 MHz and are reported as parts per million (ppm) using CD_3_CN (*δ*_H_ 1.94 ppm) as an internal reference. ^13^C NMR spectra were recorded at 298 K using a Brüker AV-400 spectrometer at a frequency of 100 MHz and are reported as parts per million (ppm) using CD_3_CN (*δ*_H_ 1.94 ppm) as an internal reference. The electronic spray ionization (ESI) mass spectra were obtained using an LCT Premier XE mass spectrometer.

### Synthesis of hetero[6]rotaxane **8**

Compound **1** (0.128 g, 0.10 mmol), BPP34C10 (0.057 g, 0.10 mmol), DB24C8 (0.118 g, 0.25 mmol), B21C7 (0.100 g, 0.26 mmol) and compound **5** (0.089 g, 0.23 mmol) were dissolved in CH_3_CN (3.0 mL) and stirred under an argon atmosphere at room temperature for 2 h. Then, Cu(CH_3_CN)_4_PF_6_ (0.021 g, 0.05 mmol) was added to the mixture and stirred under an argon atmosphere at room temperature for 48 h. Then, 15 mL of deionized water was added into the mixture and the mixture was extracted using CH_2_Cl_2_ (3 × 15 mL). The organic layers were collected, dried over Na_2_SO_4_ and concentrated under reduced pressure. After removal of the solvent, the crude product was purified by chromatography on a silica gel column (CH_2_Cl_2_/methanol = 30/1) to generate compound **8** (0.208 g, 49.4%) as a red solid. ^1^H NMR (400 MHz, CD_3_CN, 298 K) *δ* 8.82 (d, *J* = 6.3 Hz, 4H), 7.81 (d, *J* = 8.6 Hz, 6H), 7.53 (d, *J* = 8.0 Hz, 12H), 7.46 (d, *J* = 8.0 Hz, 4H), 7.37 (s, 12H), 7.25 (d, *J* = 8.4 Hz, 4H), 6.99–6.91 (m, 12H), 6.87–6.82 (m, 9H), 6.82–6.75 (m, 9H), 6.72 (d, *J* = 8.3 Hz, 4H), 5.77 (s, 4H), 4.95 (s, 4H), 4.85–4.77 (m, 4H), 4.58–4.52 (m, 4H), 4.39–4.33 (m, 4H), 4.31 (t, *J* = 7.0 Hz, 4H), 4.24 (d, *J* = 6.9 Hz, 4H), 4.21 (d, *J* = 4.8 Hz, 4H), 4.18–4.13 (m, 4H), 4.07–3.96 (m, 16H), 3.88–3.81 (m, 8H), 3.77 (d, *J* = 5.5 Hz, 16H), 3.66–3.56 (m, 66H), 3.50–3.44 (m, 16H), 3.37 (m, *J* = 10.5, 6.3 Hz, 8H), 1.82–1.77 (m, 4H), 1.55–1.49 (m, 4H). ^13^C NMR (100 MHz, CD_3_CN, 298 K) *δ* 158.6, 151.5, 147.8, 147.0, 146.6, 145.7, 145.2, 142.6, 133.8, 133.5, 132.6, 130.7, 129.8, 129.7, 129.1, 128.8, 128.4, 124.7, 123.6, 123.4, 121.4, 120.9, 120.9, 114.2, 114.1, 113.5, 112.0, 111.7, 70.8, 70.6, 70.3, 70.3, 70.0, 69.8, 69.7, 69.3, 69.2, 69.0, 68.9, 68.6, 67.9, 67.6, 67.5, 67.1, 60.9, 50.5, 50.4, 49.4, 46.6, 29.3, 29.1, 29.0, 28.6, 25.7, 25.7, 25.2. HRMS (ESI) (*m*/*z*): [M-2PF_6_]^2+^ calcd for C_184_H_248_F_24_N_12_O_42_P_4_/2: 1939.8131, found: 1939.7592; [M-3PF_6_]^3+^ calcd for C_184_H_248_F_18_N_12_O_42_P_3_/3: 1244.8872, found: 1244.8542; [M-4PF_6_]^4+^ calcd for C_184_H_248_F_12_N_12_O_42_P_2_/4: 897.4242, found: 897.3967; [M-5PF_6_]^5+^ calcd for C_184_H_248_F_6_N_12_O_42_P/5: 688.9464, found: 688.9200; [M-6PF_6_]^6+^ calcd for C_184_H_248_N_12_O_42_/6: 549.9612, found: 549.9410.

### Synthesis of **C1**

Compound **1** (0.088 g, 0.07 mmol), compound **5** (0.064 g, 0.17 mmol) and Cu(CH_3_CN)_4_PF_6_ (0.012 g, 0.04 mmol) were dissolved in CH_3_CN (3.0 mL) and stirred under an argon atmosphere at room temperature for 48 h. Then, 20 mL of deionized water was added into the mixture and extracted using CH_2_Cl_2_ (3 × 20 mL). The collected organic layers were dried over Na_2_SO_4_ and concentrated under reduced pressure. After removal of the solvent, the crude product was purified by chromatography on a silica gel column (CH_2_Cl_2_/methanol = 10/1) to give compound **C1** (0.052 g, 37.0%) as a yellow solid. ^1^H NMR (400 MHz, CD_3_CN, 298 K) *δ* 8.96 (d, *J* = 6.9 Hz, 4H), 8.41 (d, *J* = 6.9 Hz, 4H), 7.90 (s, 2H), 7.56 (q, *J* = 8.4 Hz, 8H), 7.46 (s, 10H), 7.42 (d, *J* = 8.5 Hz, 3H), 7.08 (d, *J* = 8.6 Hz, 4H), 5.85 (s, 4H), 5.15 (s, 3H), 4.36 (t, *J* = 7.1 Hz, 4H), 4.26 (s, 4H), 4.20 (s, 3H), 4.16 (s, 4H), 3.07–2.98 (m, 4H). ^13^C NMR (100 MHz, CD_3_CN, 298 K) *δ* 159.1, 150.1, 145.3, 133.6, 132.0, 131.6, 130.9, 130.2, 129.7, 129.4, 129.4, 128.7, 127.2, 122.3, 114.7, 63.7, 61.0, 51.2, 50.8, 50.2, 49.5, 47.4, 29.1, 25.0, 24.9, 24.9. HRMS (ESI) (*m*/*z*): [M-2PF_6_]^2+^ calcd for C_72_H_88_F_24_N_12_O_2_P_4_/2: 866.29, found: 866.26; [M-3PF_6_]^3+^ calcd for C_72_H_88_F_18_N_12_O_2_P_3_/3: 529.21, found: 529.20; [M-4PF_6_]^4+^ calcd for C_72_H_88_F_12_N_12_O_2_P_2_/4: 360.66, found: 360.66; [M-6PF_6_]^6+^ calcd for C_72_H_88_N_12_O_2_/6: 192.12, found: 192.18.

## Conclusions

In conclusion, by precisely programming the association constants, steric hindrance and size-matching of different macrocyclic rings and their corresponding guest molecules, we have developed and constructed a novel six-component self-sorting system. The self-sorting strategy as a key tool ensures this highly selective self-assembly process in a multi-component system and gives rise to the formation of specific rotaxane precursors. Based on this well-organized self-sorting process, a novel hetero[6]rotaxane **8** with three different crown ether macrocycles, a BPY^2+^ recognition site and two secondary ammonium recognition sites was efficiently constructed *via* a classical CuAAC stoppering reaction. Such work has not only successfully developed a novel hetero[6]rotaxane **8**, which will enrich the family of mechanically interlocked molecules, but also provides a good supplement for the self-sorting concept that can be used for the construction of complex supramolecular systems and materials with increasing complexity both in terms of structure and function.
