# Supplementary material for: One-pot synthesis of hetero[6]rotaxane bearing three different kinds of macrocycle through a self-sorting process
Source: Chem Sci. 2017 Aug 4;8(10):6777–83. doi: 10.1039/c7sc03232c (PMC5643886; doi:10.1039/c7sc03232c)
Supplement: Supplementary file 1 [file SC-008-C7SC03232C-s001.pdf]

Electronic Supplementary Material (ESI) for Chemical Science.
This journal is © The Royal Society of Chemistry 2017

Supporting Information

One-pot Synthesis of Hetero[6]rotaxane Bearing Three Different kinds of Macrocycles through a Self-Sorting Process

Si-Jia Rao, Qi Zhang, Ju Mei, Xu-Hao Ye, Chuan Gao, Qiao-Chun Wang, Da-Hui Qu* and He Tian

Key Laboratory for Advanced Materials and Institute of Fine Chemicals, School of Chemistry and molecular Engineering, East China University of Science and Technology, 130 Meilong Road, Shanghai, 200237 (China).

Contents

1. General Information..2
2. Synthesis..3
Preparation of compound S2:..4
Preparation of compound S3:..4
Preparation of compound S4:..5
Preparation of compound 1:..5
3. Characterization Data and Their Original Spectra..6
References:..24

1. General Information

Chemicals were purchased from Adamas-beta® and used as received unless otherwise stated. Solvents were reagent grade pure, which were dried and distilled prior to use according to standard procedures. All reactions were carried out under an atmosphere of dry nitrogen unless otherwise stated. NMR experiments (^{1}H NMR, ^{13}C NMR, ^{1}H-^{1}H COSY and ^{1}H-^{1}H NOESY) were measured on a Brüker AV-400 spectrometer. The electronic spray ionization (ESI) mass spectra were tested on a LCT Premier XE mass spectrometer.

2. Synthesis

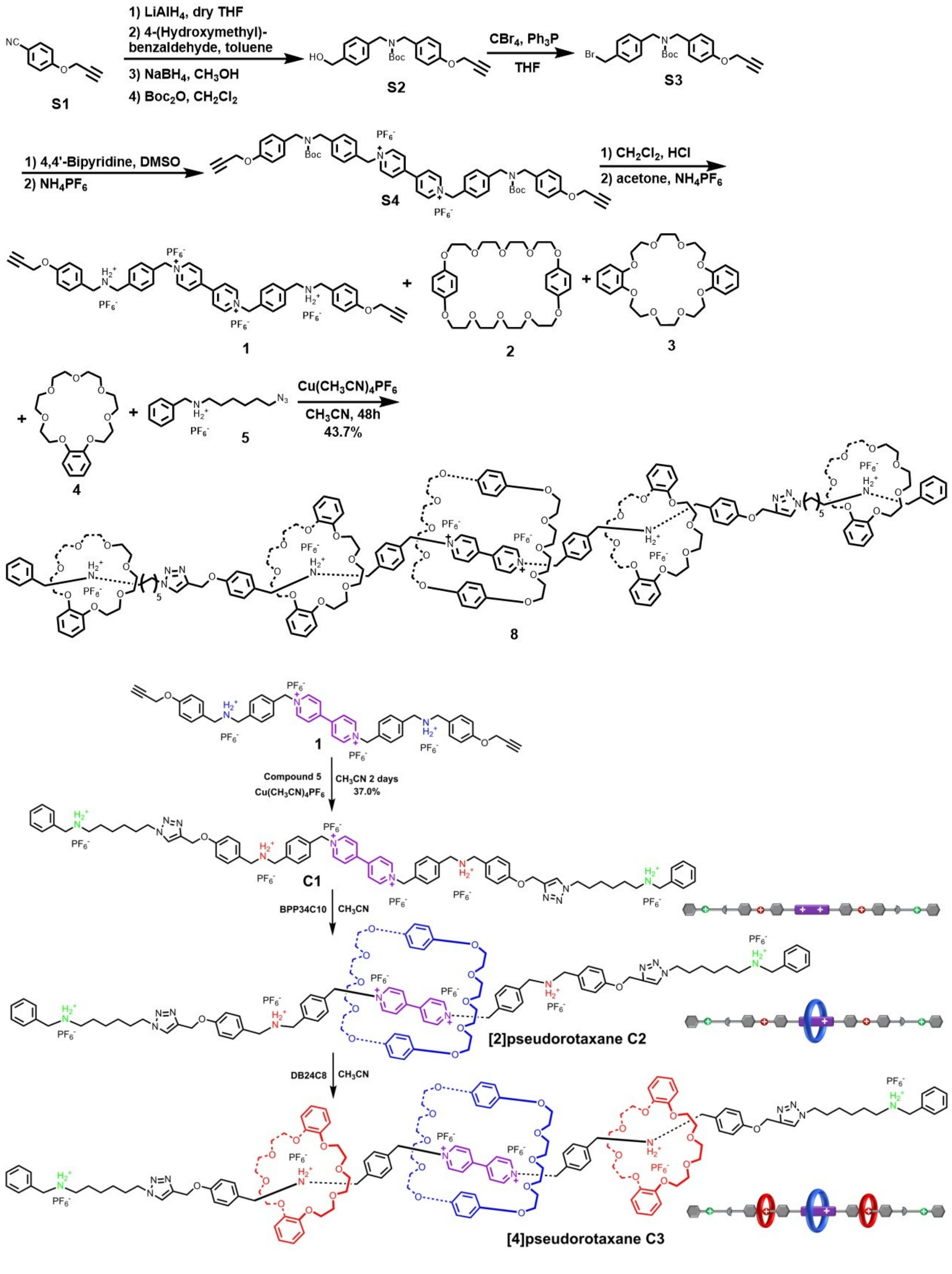

Scheme S1. The synthetic routes of compound **C1** and hetero[6]rotaxane **8** and the stepwise formation of pseudo[2]rotaxane **C2** and pseudo[4]rotaxane **C3**.

Compound **S1**[S1], compound **2** [S2], compound **3** [S3], compound **4** [S3], and compound **5** [S3] were synthesized according to the previously reported literatures.

Compound **S2:** Compound **S1** (0.852 g, 5.42mmol) was dissolved in dry THF and lithium aluminium hydride (1.875 g, 54.21 mmol) was slowly added in portions to the mixture under ice bath. The mixture was stirred at room temperature for 5 hours. Then 2.0 mL water and 2.0 mL 15 % aqueous sodium hydroxide were added slowly to the mixture to quench the reaction. The mixture was filtrated and the solid was washed by THF (30 mL). The collected organic phase, it was washed with water (3 × 30 mL). The organic layers were combined and dried over Na_2SO_4 and finally concentrated under reduced pressure. The crude product and 4-(hydroxymethyl)benzaldehyde (0.453 g, 3.10 mmol) were dissolved in toluene (15 mL) and the mixture was heated and stirred under reflux in an argon atmosphere for 24 hours. After cooling, toluene was evaporated under reduced pressure. The crude product was dissolved in dry methanol and $NaBH_4$ (1.266 g, 33.27 mmol) was slowly added in portions to the mixture under ice bath. The mixture was stirred at room temperature for 5 hours. Then, 15 mL water was added slowly to the mixture to quench the reaction, After the methanol was evaporated, and the residue was extracted by CH_2Cl_2 (3 × 15 mL). The organic phase was collected and washed with water (3 × 20 mL). The combined organic layers were dried over Na_2SO_4 and concentrated under reduced pressure. The crude product and Boc_2O (2.658g, 12.18 mmol) were dissolved in CH_2Cl_2 (15mL). The mixture was stirred at room temperature for 6 hours. The organic phase was washed with water (3 × 20 mL). The collected organic layers were dried over Na_2SO_4 and concentrated under reduced pressure. After removal of the solvent, the crude product was purified by chromatography on a silica gel column (CH_2Cl_2/methanol = 100/1) to afford the compound **S2** (1.321g, 63.9 %) as a yellow oily liquid. ^1H NMR (400 MHz, $CDCl_3$) δ 7.32 (d, J = 8.0 Hz, 2H), 7.17 (d, J = 6.7 Hz, 4H), 6.94 (dd, J = 6.6, 4.8 Hz, 2H), 4.68 (d, J = 2.4 Hz, 4H), 4.32 (dd, J = 29.9, 16.4 Hz, 4H), 2.53 (t, J = 2.3 Hz, 1H), 1.49 (s, 9H). ^{13}C NMR (100 MHz, $CDCl_3$) δ 156.83, 156.0, 139.9, 130.9, 127.1, 114.9, 80.1, 78.5, 75.5, 65.0, 55.8, 28.4. HRMS (ESI) (m/z): $[M+Na]^+$ calcd for $C_{23}H_{27}NO_4Na$: 404.1838, found: 404.1837.

Compound **S3**: Compound **S2** (0.348 g, 0.91 mmol) was dissolved in dry THF (5.0 mL) and the solution was added to CBr_4 (0.460 g, 1.37mmol) and Ph_3P (0.493 g, 1.82 mmol) and stirred in ice bath for 2 hours. Then, 25 mL saturated brines was added and the mixture was extracted by CH_2Cl_2 (3 × 30 mL). The organic layers were collected and dried over Na_2SO_4 and concentrated under reduced pressure. After removal of the solvent, the crude product was purified by chromatography on a silica gel column (CH_2Cl_2/methanol = 150/1) to yield the compound **S3** (0.260g, 64.3 %) as a yellow oily liquid. ^1H NMR (400 MHz, $CDCl_3$) δ 7.35 (d, J = 8.1 Hz, 2H), 7.16 (s, 4H), 6.97 – 6.90 (m, 2H), 4.69 (d, J = 2.3 Hz, 2H), 4.50 (s, 2H), 4.42 – 4.21 (m, 4H), 2.53 (t, J = 2.4 Hz, 1H), 1.49 (s, 9H). ^{13}C NMR (100 MHz, $CDCl_3$) δ 156.8, 155.9, 136.7, 129.2, 114.9, 80.2, 78.5, 75.5, 55.8, 33.3, 28.4. HRMS (ESI) (m/z): $[M+Na]^+$ calcd for $C_{23}H_{26}NO_3BrNa$: 466.0944, found: 466.0989.

Compound **S4:** The mixture of compound **S3** (0.242 g, 0.55 mmol) and 4, 4'-bipyridine (0.036 g, 0.22 mmol) was dissolved in dimethyl sulphoxide (DMSO) were stirred under reflux for 14 hours. Then, the mixture was added to 15.0 mL saturated NH_4PF_6 solution. The resultant mixture was filtrated and the solid was washed by deionized water (30 mL). Then, give the compound **S4** (0.175g, 64.4 %) was afforded as a yellow solid. ^1H NMR (400 MHz, CD_3CN) δ 8.95 (d, J = 6.9 Hz, 4H), 8.37 (d, J = 6.8 Hz, 4H), 7.47 – 7.40 (m, 4H), 7.32 (d, J = 8.1 Hz, 4H), 7.17 (d, J = 8.7 Hz, 4H), 6.90 (d, J = 8.7 Hz, 4H), 5.79 (s, 4H), 4.70 (d, J = 2.4 Hz, 4H), 4.40 (s, 4H), 4.34 (s, 4H), 2.79 (t, J = 2.4 Hz, 2H), 1.43 (s, 18H). ^{13}C NMR (100 MHz, CD_3CN) δ 157.6, 156.5, 151.1, 146.4, 132.1, 130.3, 129.8, 129.4, 128.3, 115.6, 80.6, 79.6, 76.7, 65.3, 56.4, 28.4. HRMS (ESI) (m/z): $[M-PF_6]^+$ calcd for $C_{56}H_{60}F_6N_4O_6P$: 1029.4149, found: 1029.4155.

Compound **1**: Compound **S4** (0.194 g, 0.165 mmol) was dissolved in CH_2Cl_2 (3.0 mL) and added to HCl (12 mol/L, 2.0 mL) and stirred for 8 hours. Then, 15.0 mL saturated NH_4PF_6 solution was added. The crude product was washed by 30 mL deionized water to yield the compound **1** (0.182g, 87.0 %) as a yellow solid. ^1H NMR (400 MHz, CD_3CN) δ 8.96 (d, J = 6.9 Hz, 4H), 8.39 (d, J = 6.9 Hz, 4H), 7.56 (q, J = 8.3 Hz, 8H), 7.43 (d, J = 8.7 Hz, 5H), 7.32 (d, J = 8.5 Hz, 4H), 7.04 (d, J = 8.7 Hz, 4H), 5.84 (s, 4H), 4.77 (d, J = 2.4 Hz, 4H), 4.24 (s, 4H), 4.19 (s, 4H), 2.83 (t, J = 2.4 Hz, 2H). ^{13}C NMR (100 MHz, CD_3CN) δ 158.1, 150.1, 145.3, 133.6, 132.0, 131.6, 130.9, 129.4, 127.2, 122.7, 114.8, 78.0, 75.8, 63.7, 55.2, 50.7, 50.1. HRMS (ESI) (m/z): $[M-PF_6]^+$ calcd for $C_{46}H_{46}F_{18}N_4O_2P_3$: 1121.2541, found: 1121.2543.

The corresponding original spectra of the above compounds are displayed as Figures S11-35 in the following section.

3. Characterization Data and Their Original Spectra

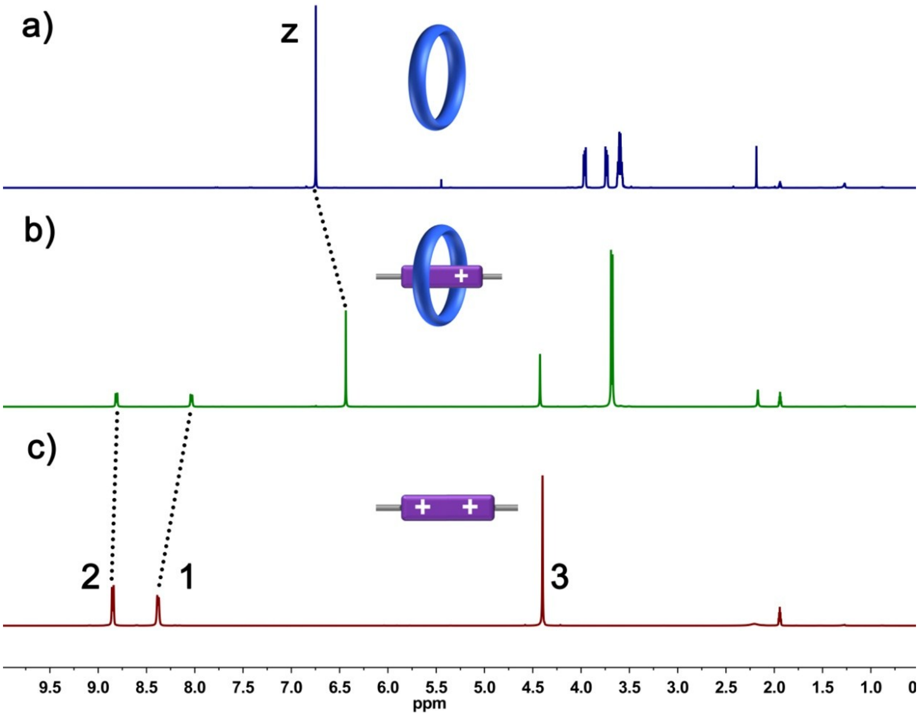

Figure S1. Partial ^1H NMR spectra (400 MHz, 298 K, CD_3CN) of a) BPP34C10, b) BPP34C10⊃BPY^{2+} and c) BPY^{2+}.

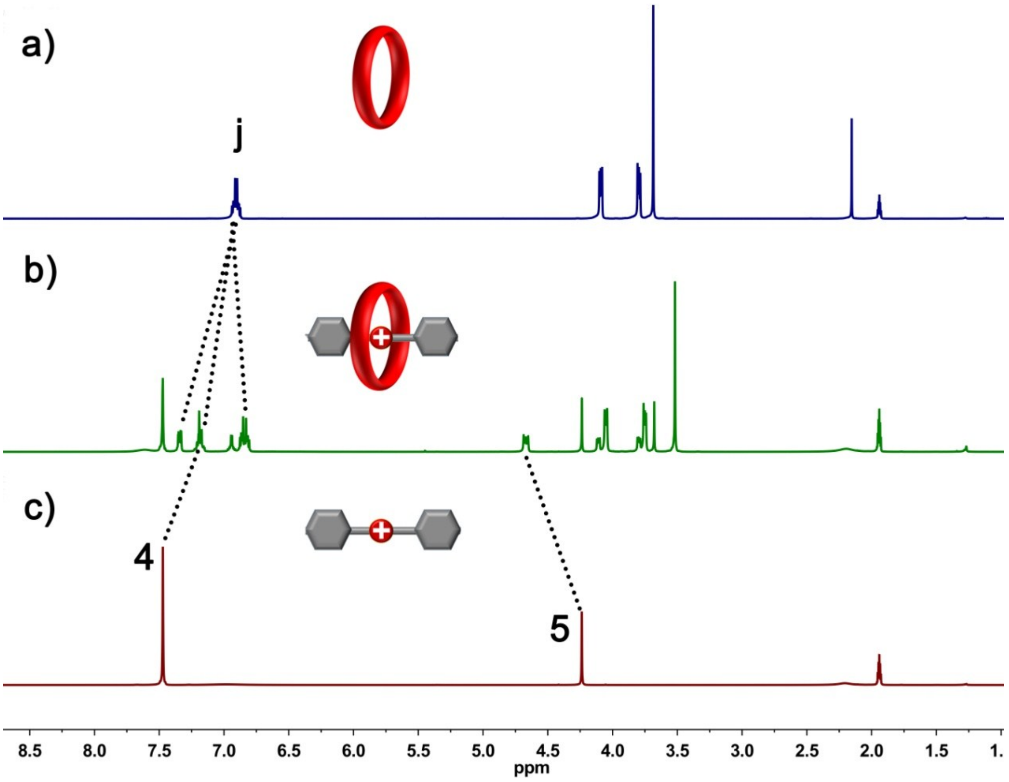

Figure S2. Partial ^1H NMR spectra (400 MHz, 298 K, CD_3CN) of a) DB24C8, b) DB24C8⊃DBA and c) DBA.

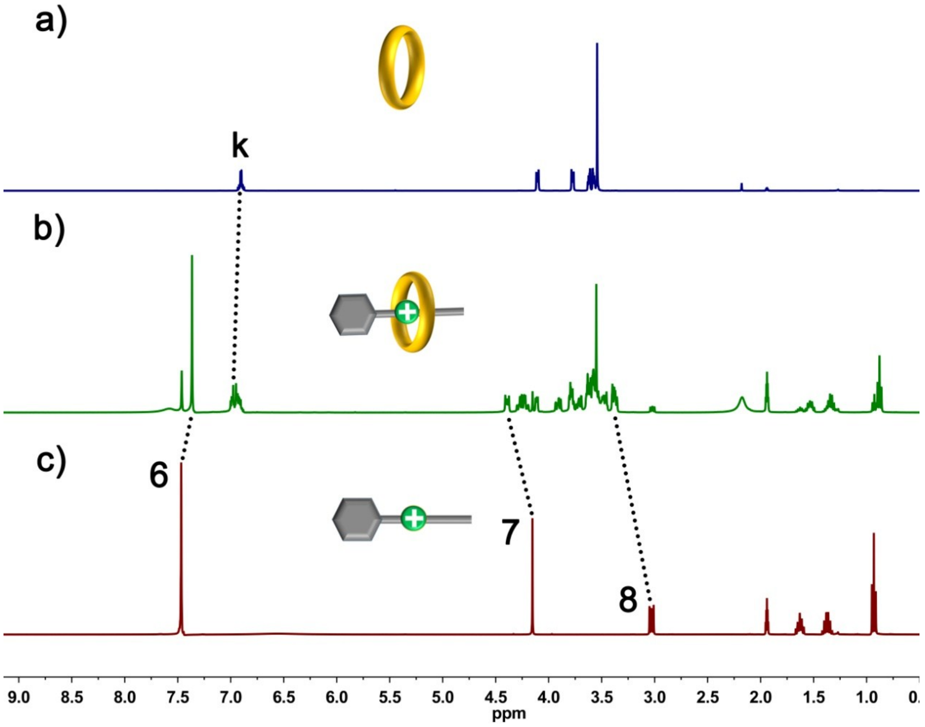

Figure S3. Partial ^{1}H NMR spectra (400 MHz, 298 K, CD_3CN) of a) B21C7, b) B21C1⊃BAA and c) BBA

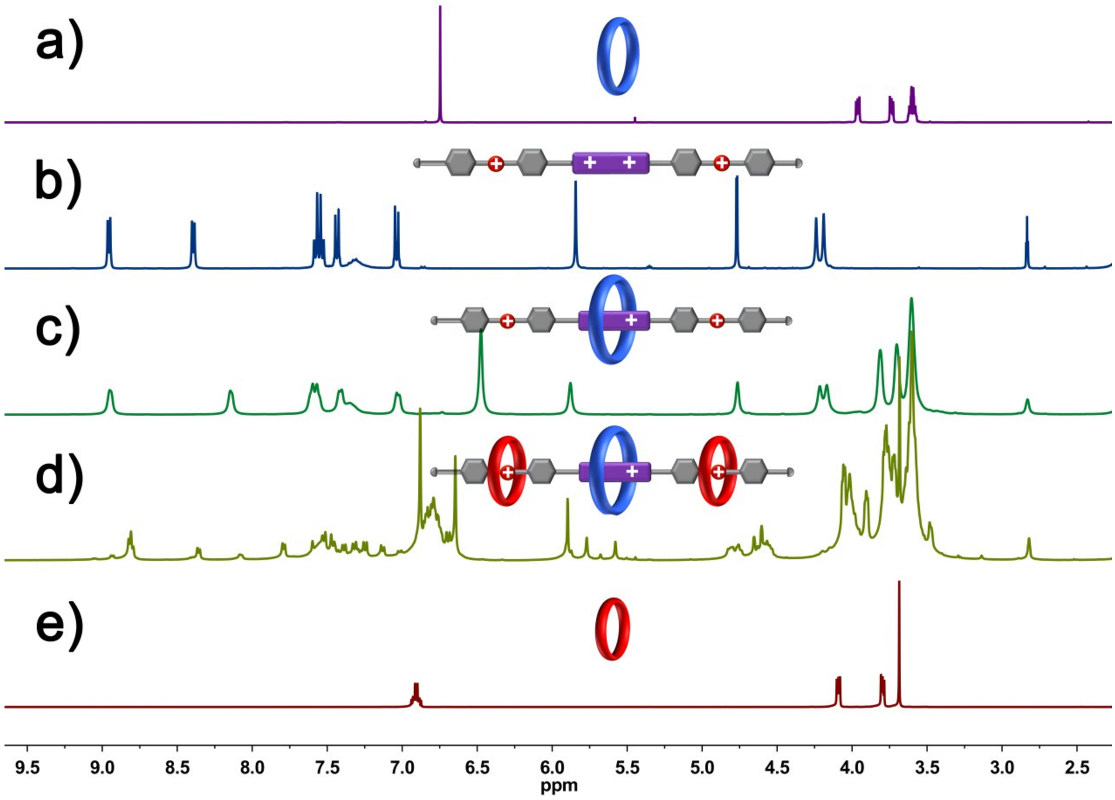

Figure S4. Partial ^{1}H NMR spectra (400 MHz, 298 K, CD_3CN) of a) BPP34C10, b) compound **1**, c) 1:1 mixture of **1**, BPP34C10, d) 1:1:2 mixture of **1**, BPP34C10, DB24C8 and e) DB24C8.

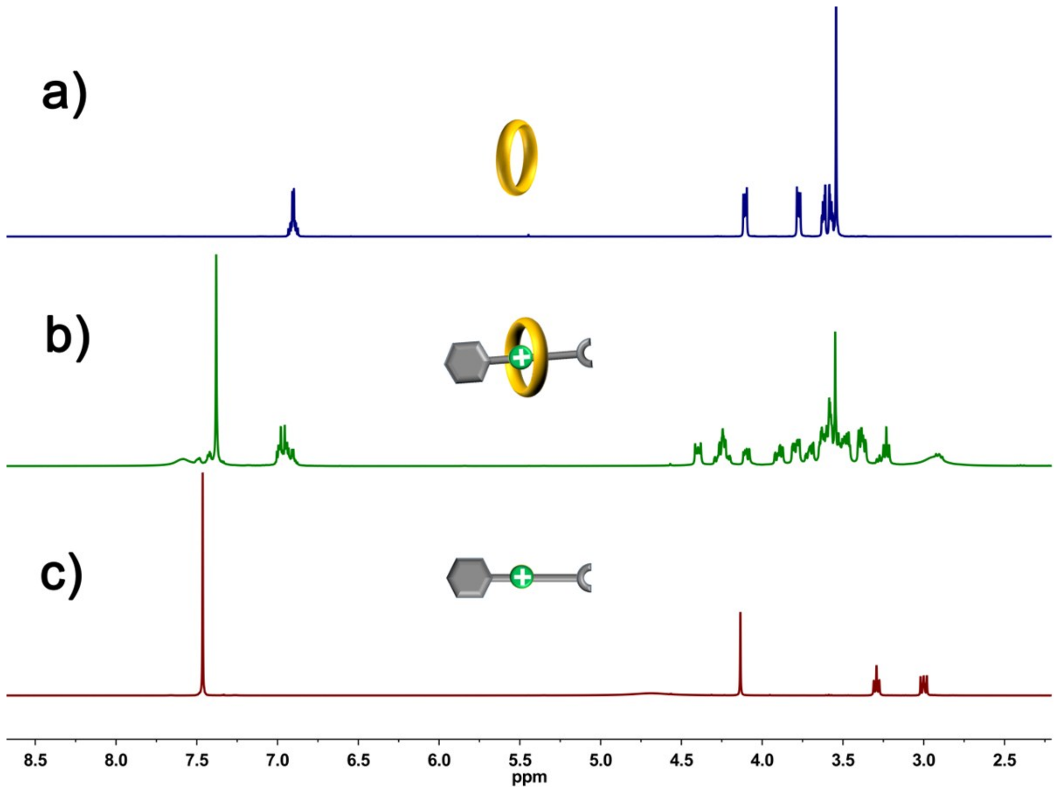

Figure S5. Partial ^{1}H NMR spectra (400 MHz, 298 K, CD_3CN) of a) B21C7, b) 1:1:2 mixture of **5**, B21C7 and c) compound **5**.

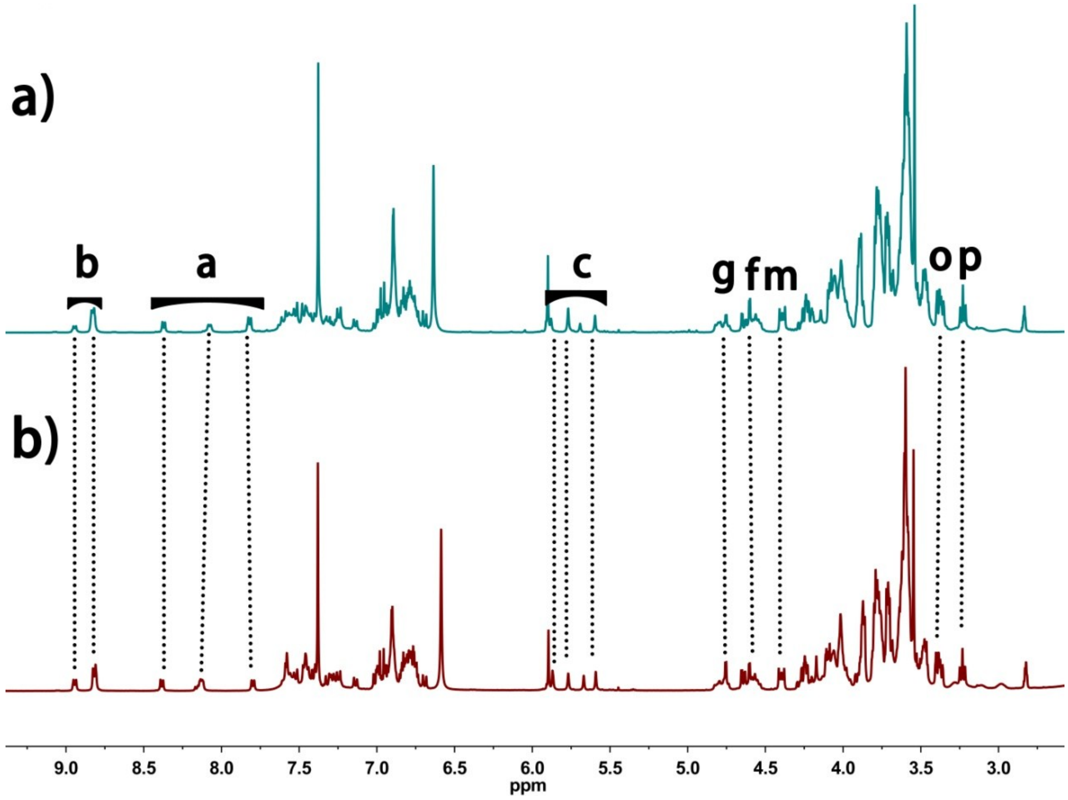

Figure S6. Partial ^{1}H NMR spectra (400 MHz, 298 K, CD_3CN) of a) equal molar mixture pseudo[4]rotaxane **6** and [2]semi-rotaxane **7**, and b) one pot mixture of compound **1**, BPP34C10, DB24C8, B21C7 and compound **5**.

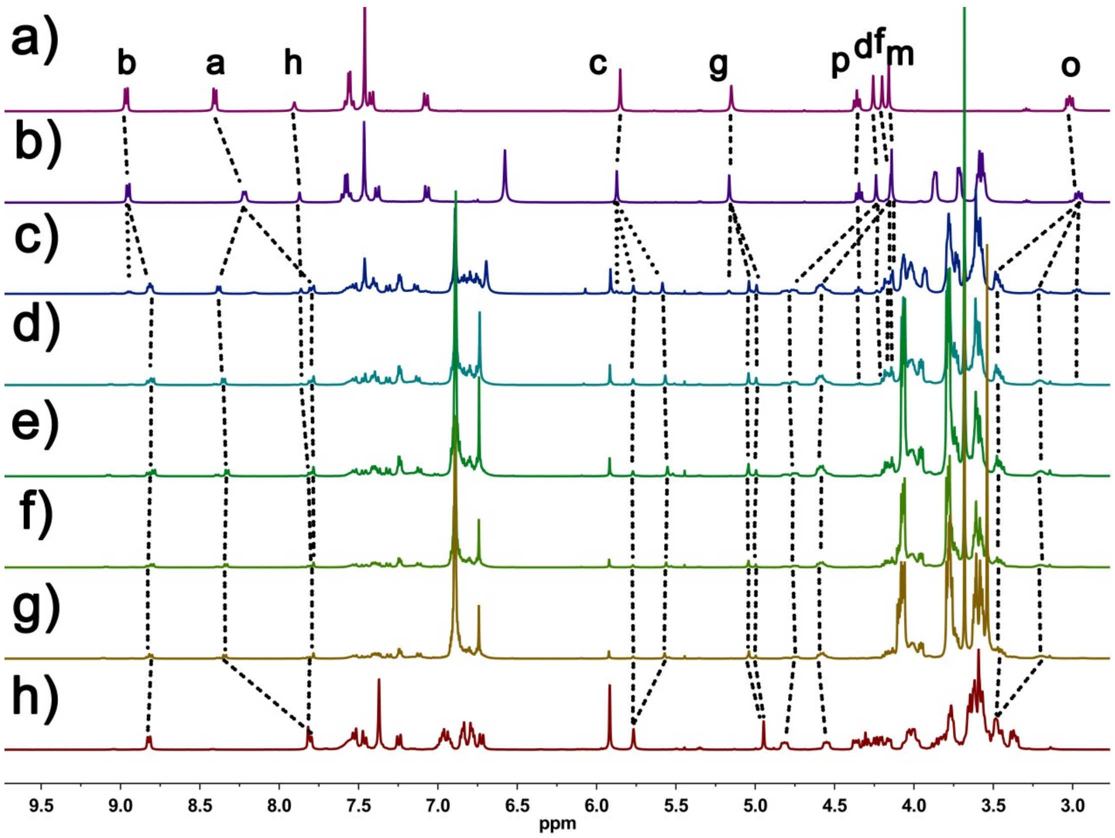

Figure S7. Partial ^{1}H NMR spectra (400 MHz, 298 K, CD_3CN) of a) compound **C1**, b) 1:1 mixture of **C1** and BPP34C10, c) 1:1:2 mixture of **C1**, BPP34C10 and DB24C8, d) 1:1:4 mixture of **C1**, BPP34C10 and DB24C8, e) 1:1:6 mixture of **C1**, BPP34C10 and DB24C8, f) 1:1:6:2 mixture of **C1**, BPP34C10, DB24C8 and B21C7, g) 1:1:6:6 mixture of **C1**, BPP34C10, DB24C8 and B21C7, and h) hetero[6]rotaxane **8**. The DB24C8 molar ratio was increasing from 2.0 equivalents to 6.0 equivalents (Figure S7c–S7e). It can illustrate that pseudo[4]rotaxane **6** was the primary species in the self-sorting process. The B21C7 molar ratio was increasing from 2.0 equivalents to 6.0 equivalents (Figure S7f–S7g) and no further change was observed upon the addition of B21C7, which indicate that the phenyl group in the terminal of the axle compound prevent the threading into the B21C7 cavity.

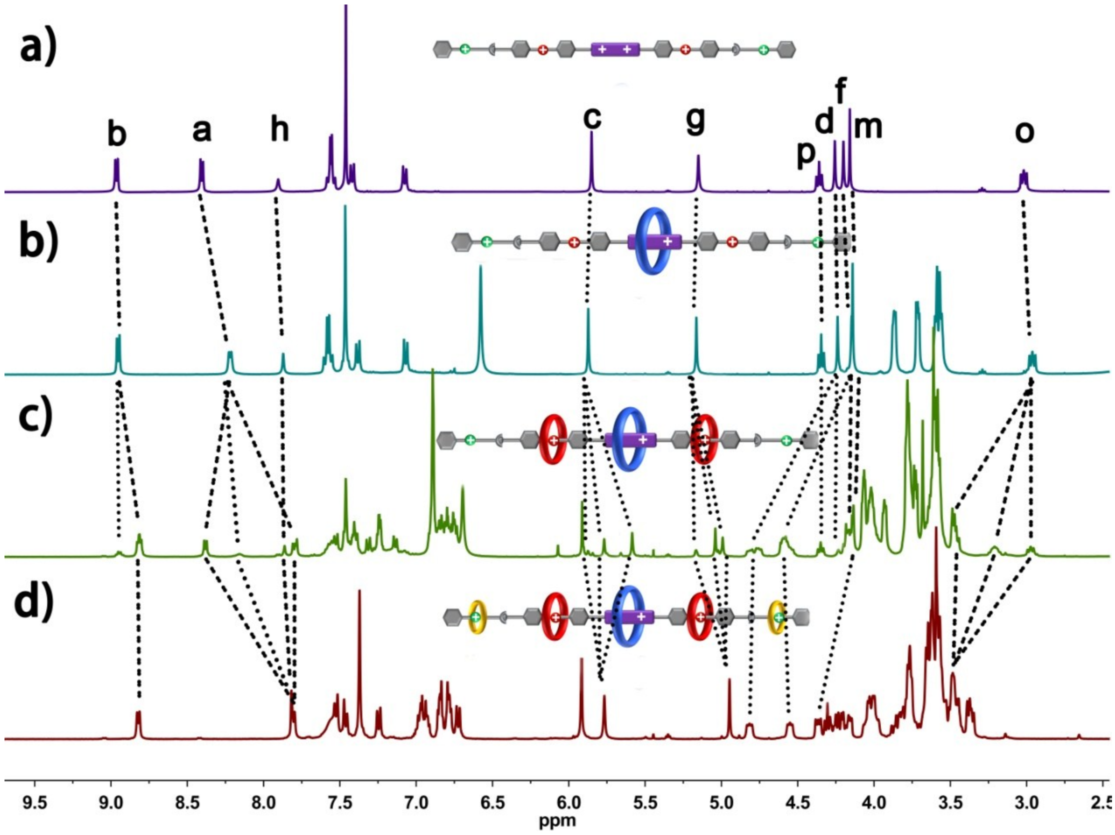

Figure S8. Partial ^{1}H NMR spectra (400 MHz, 298 K, CD_3CN) of a) compound **C1**, b) 1:1 mixture of **C1** and BPP34C10, c) 1:1:2 mixture of **C1**, BPP34C10 and DB24C8, and d) compound **8**.

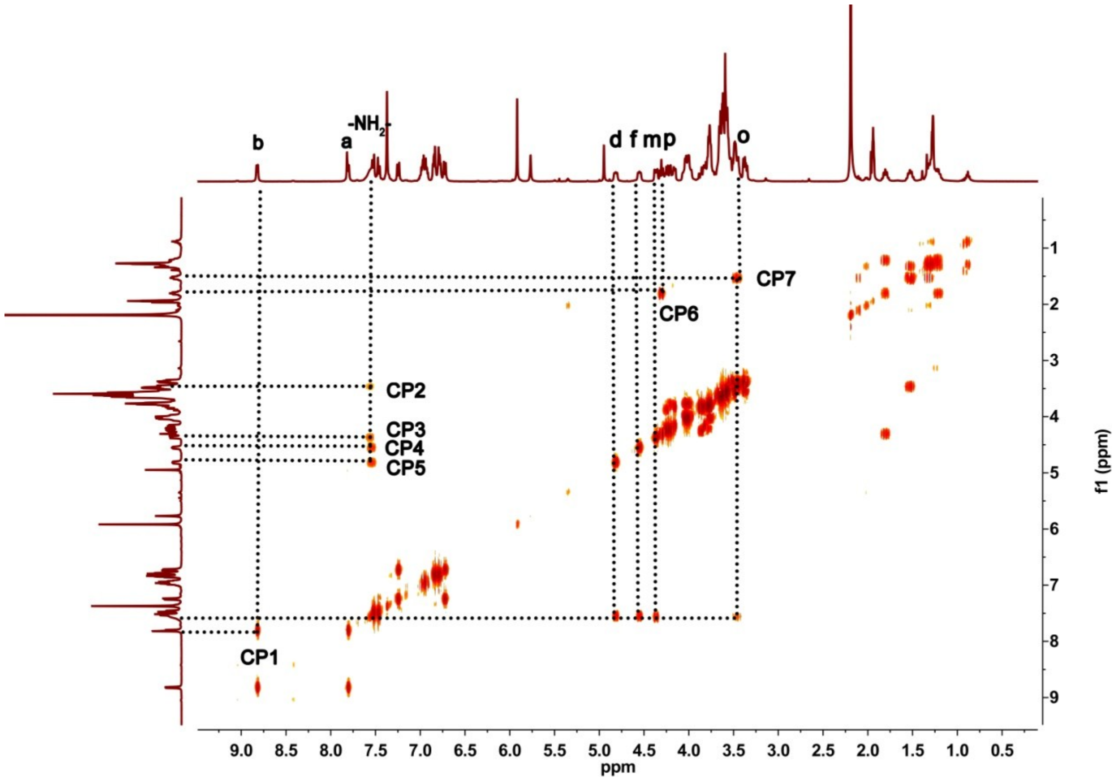

Figure S9. ^{1}H-^{1}H cosy spectrum of hetero[6]rotaxane **8** (400 MHz, 298 K, CD_3CN).

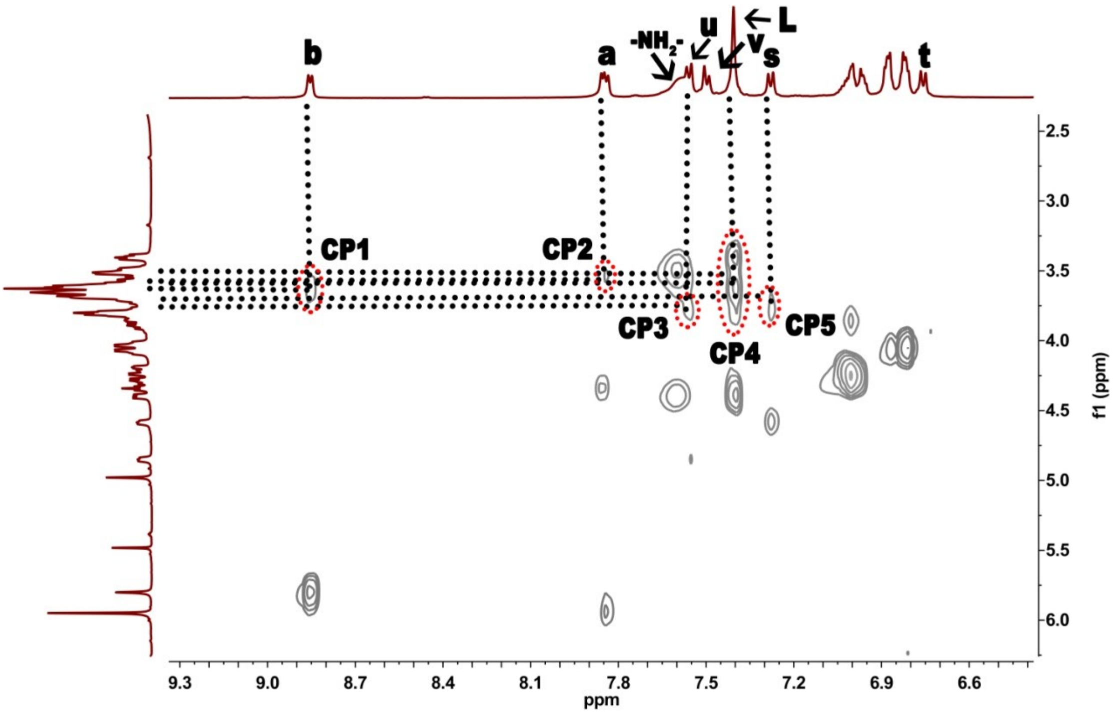

Figure S10. ^1H-^1H NOESY spectrum of hetero[6]rotaxane **8** (400 MHz, 298 K, CD_3CN).

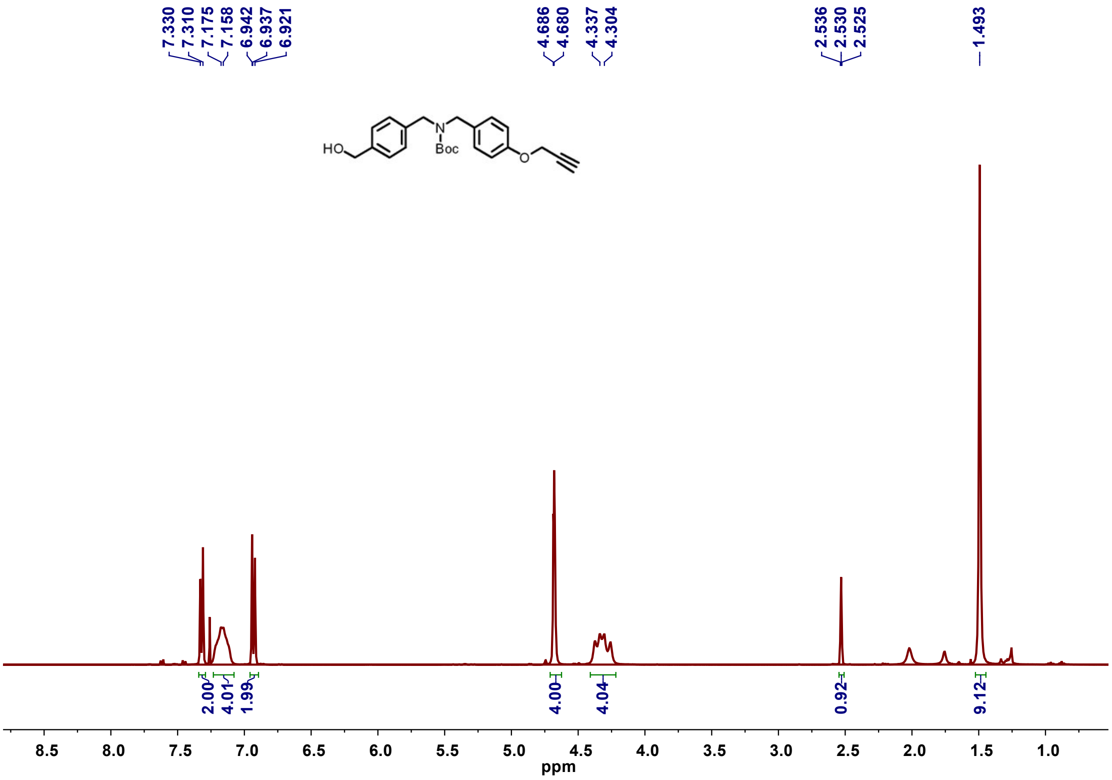

Figure S11. ^1H NMR spectrum of **S2** ($CDCl_3$, 400MHz, 298K).

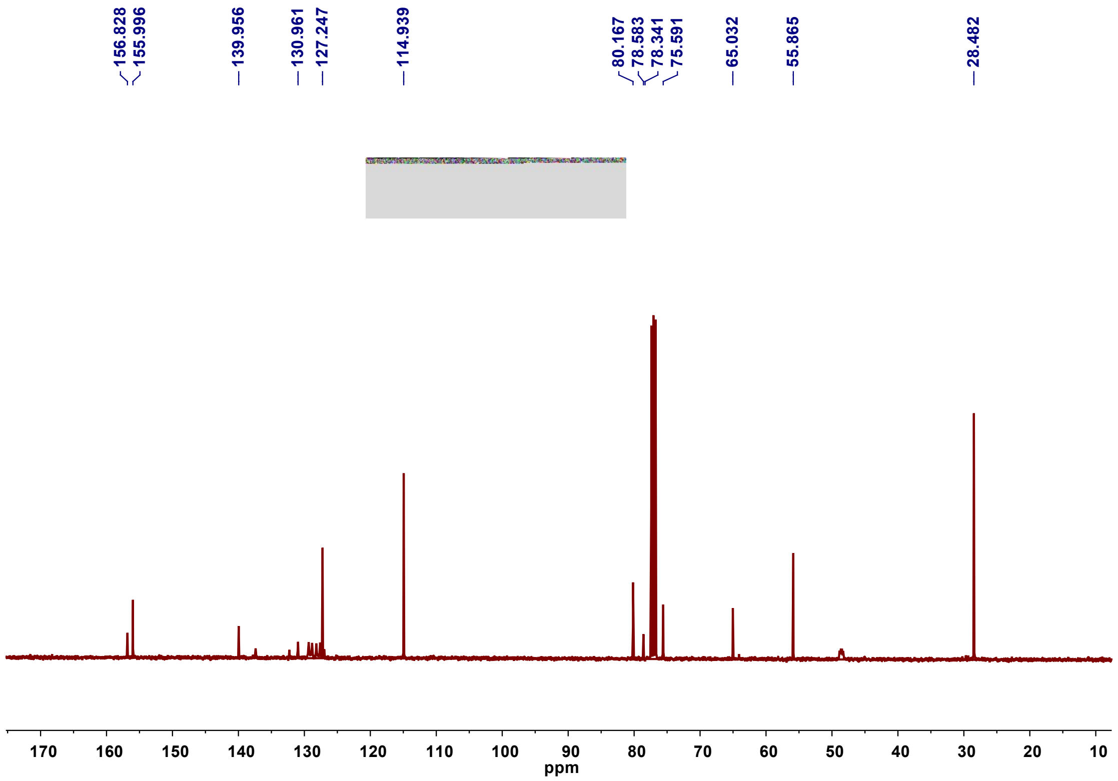

Figure S12. ^{13}C NMR spectrum of **S2** ($CDCl_3$, 100MHz, 298K).

Elemental Composition Report **Page 1**

Single Mass Analysis
Tolerance = 50.0 PPM / DBE: min = -1.5, max = 100.0
Element prediction: Off
Number of isotope peaks used for i-FIT = 3

Monoisotopic Mass, Even Electron Ions
16 formula(e) evaluated with 1 results within limits (up to 1 closest results for each mass)
Elements Used:
C: 0-23 H: 0-27 N: 0-1 O: 0-4 Na: 0-1

DH-QU ECUST institute of Fine Chem 04-May-2016
23:10:30
QDH-SJ-91 32 (0.290) Cm (32:39) 1: TOF MS ES+
1.18e+004

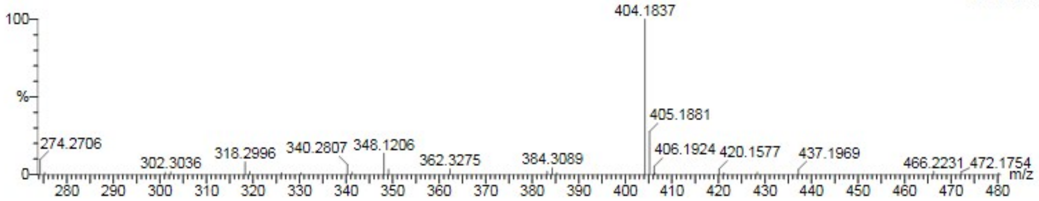

Minimum:				-1.5			
Maximum:		300.0	50.0	100.0			
Mass	Calc. Mass	mDa	PPM	DBE	i-FIT	i-FIT (Norm)	Formula
404.1837	404.1838	-0.1	-0.2	10.5	21.8	0.0	C23 H27 N O4 Na

Figure S13. ESI-mass spectrum of compound **S2** ([M+Na]$^+$: 404.1837).

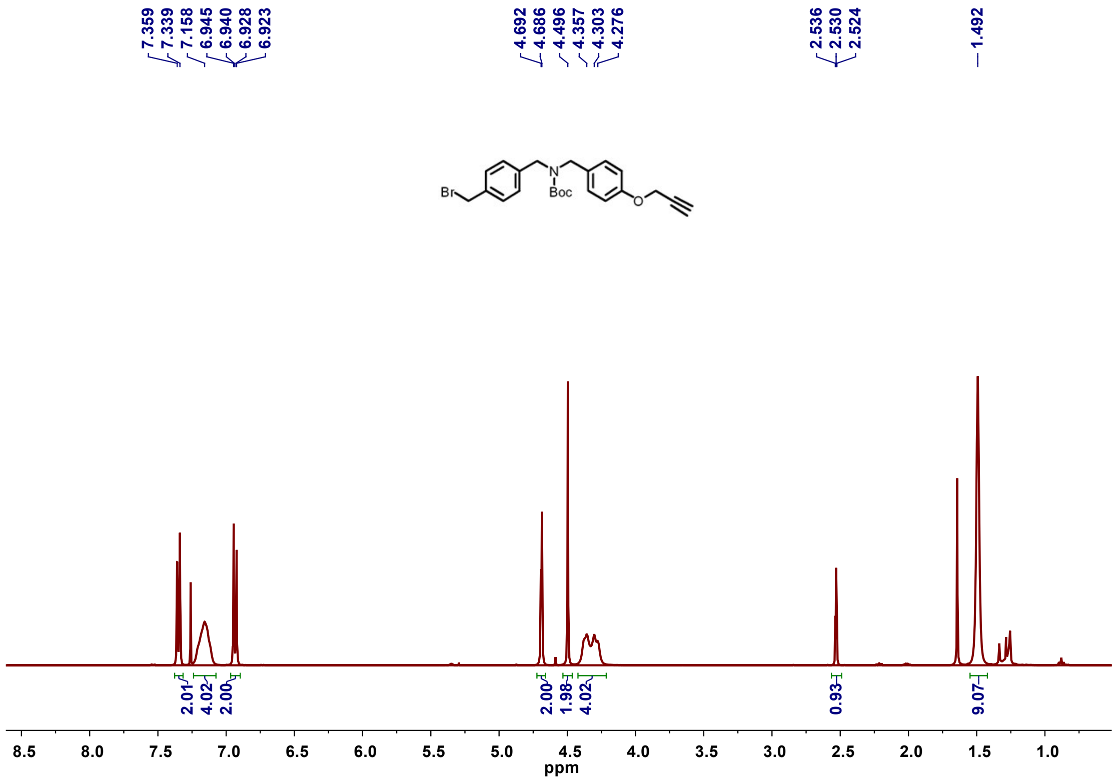

Figure S14. ^{1}H NMR spectrum of **S3** ($CDCl_3$, 400MHz, 298K).

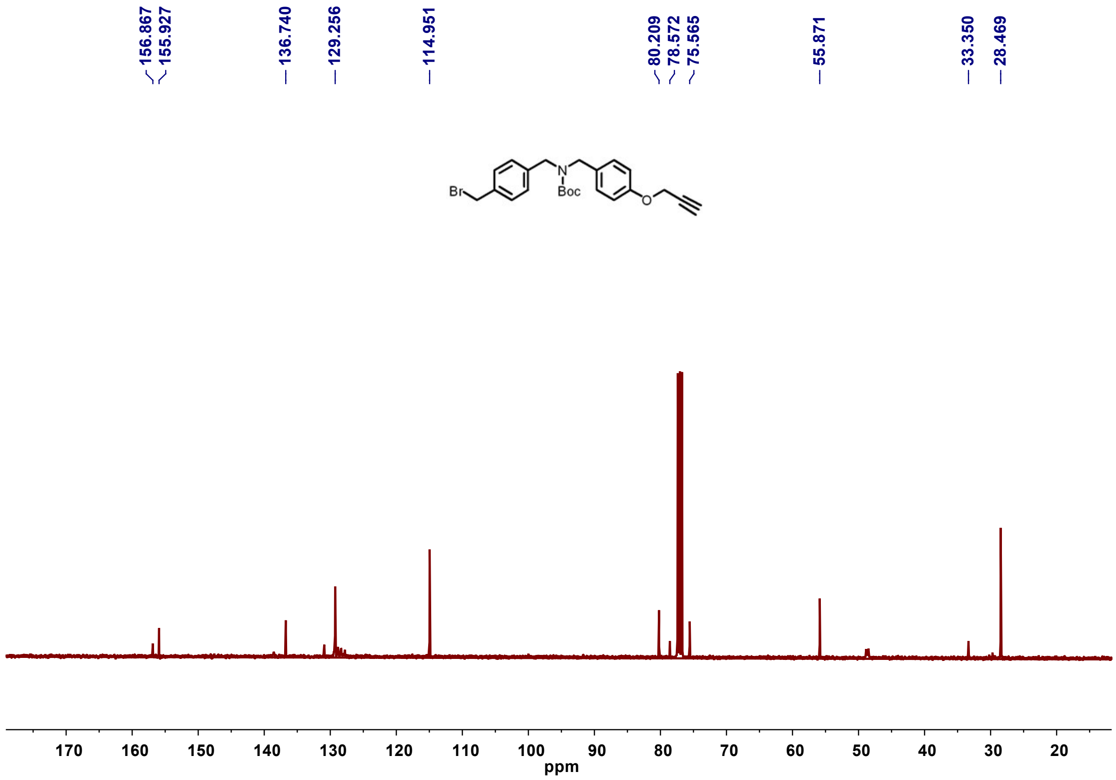

Figure S15. ^{13}C NMR spectrum of **S3** ($CDCl_3$, 100MHz, 298K)

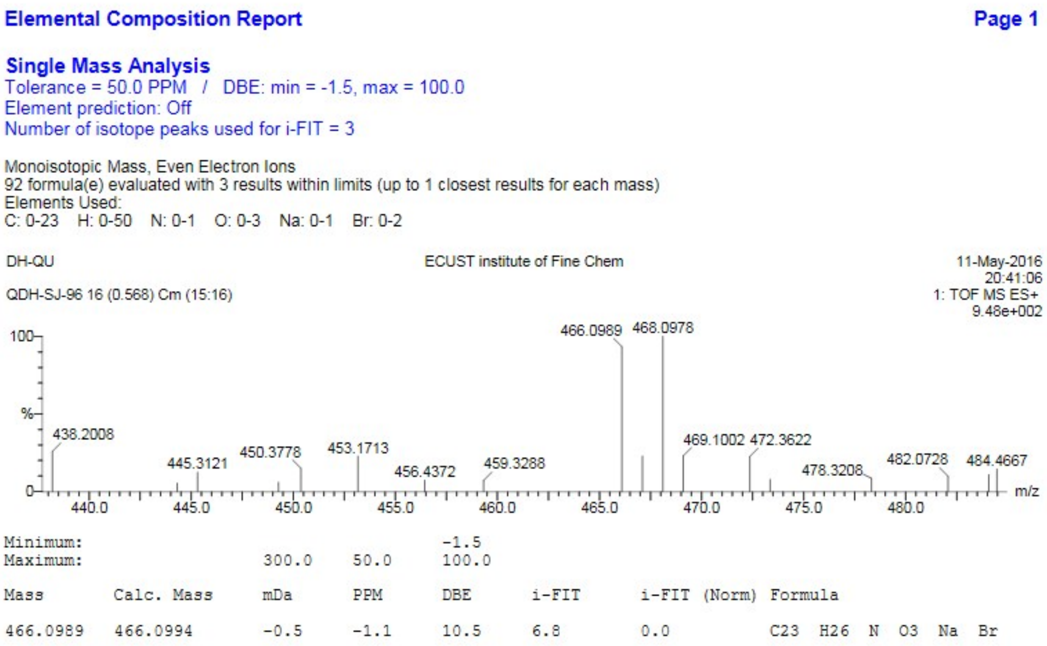
Elemental Composition Report Page 1

Single Mass Analysis
Tolerance = 50.0 PPM / DBE: min = -1.5, max = 100.0
Element prediction: Off
Number of isotope peaks used for i-FIT = 3

Monoisotopic Mass, Even Electron Ions
92 formula(e) evaluated with 3 results within limits (up to 1 closest results for each mass)
Elements Used:
C: 0-23 H: 0-50 N: 0-1 O: 0-3 Na: 0-1 Br: 0-2

DH-QU ECUST institute of Fine Chem 11-May-2016 20:41:06
QDH-SJ-96 16 (0.568) Cm (15:16) 1: TOF MS ES+ 9.48e+002

Minimum:				-1.5			
Maximum:		300.0	50.0	100.0			
Mass	Calc. Mass	mDa	PPM	DBE	i-FIT	i-FIT (Norm)	Formula
466.0989	466.0994	-0.5	-1.1	10.5	6.8	0.0	C23 H26 N O3 Na Br

Figure S16. ESI-mass spectrum of compound **S3** ([M+Na]$^{+}$: 466.0989)

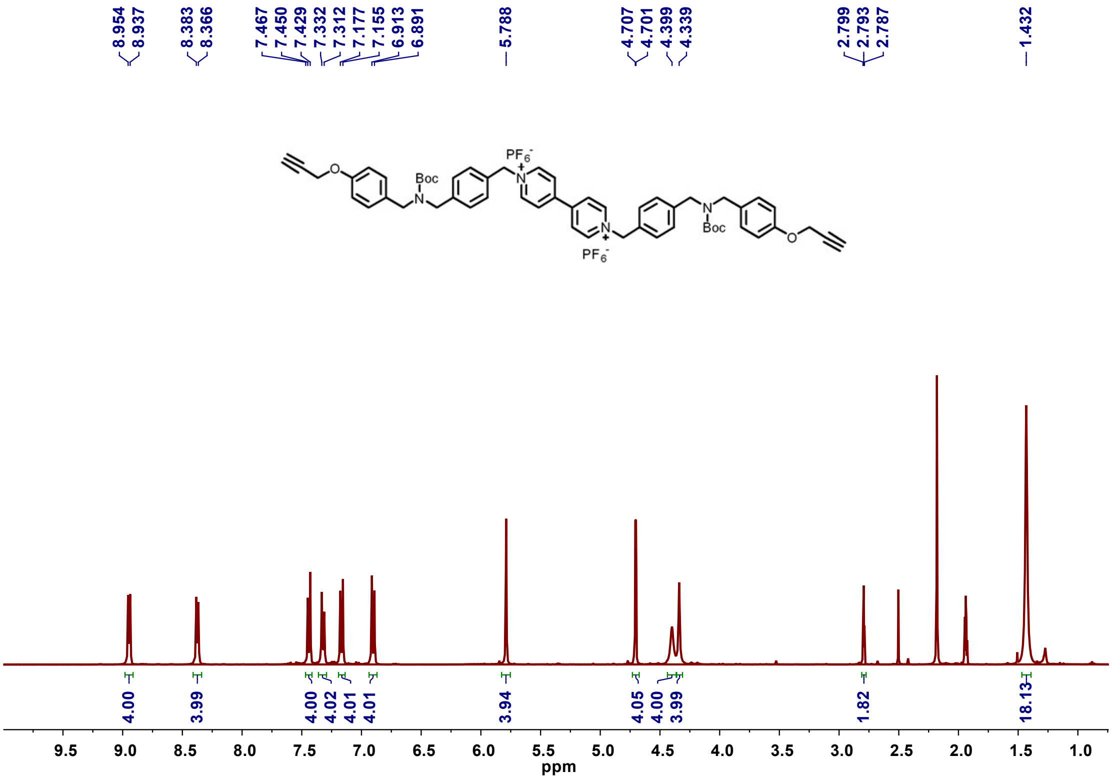

Figure S17. ^{1}H NMR spectrum of **S4** (CD_3CN, 400MHz, 298K)

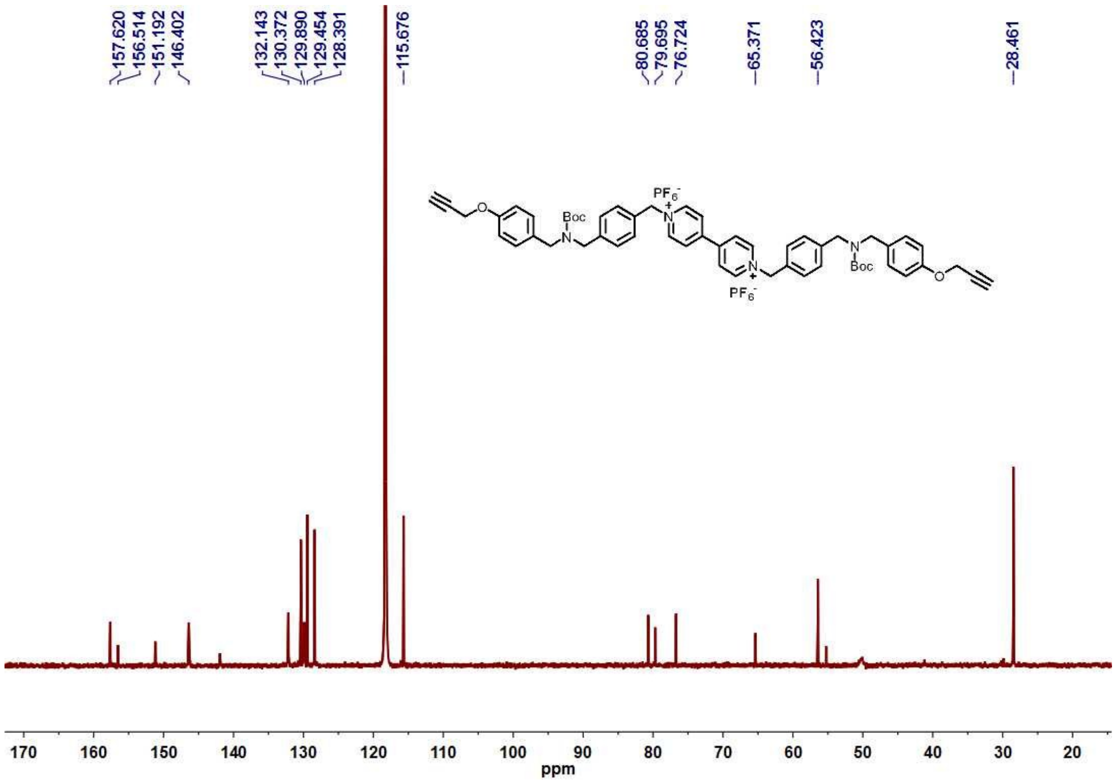

Figure S18. ^{13}C NMR spectrum of **S4** (CD_3CN, 100MHz, 298K)

Elemental Composition Report Page 1

Single Mass Analysis
Tolerance = 50.0 PPM / DBE: min = -1.5, max = 100.0
Element prediction: Off
Number of isotope peaks used for i-FIT = 3

Monoisotopic Mass, Even Electron Ions
611 formula(e) evaluated with 51 results within limits (up to 1 closest results for each mass)
Elements Used:
C: 0-60 H: 0-70 N: 0-4 O: 0-6 P: 0-1 F: 0-8

DH-QU ECUST institute of Fine Chem 13-May-2016 20:41:28

QDH-SJ-97 29 (0.988) Cm (29:32) 1: TOF MS ES+ 2.27e+002

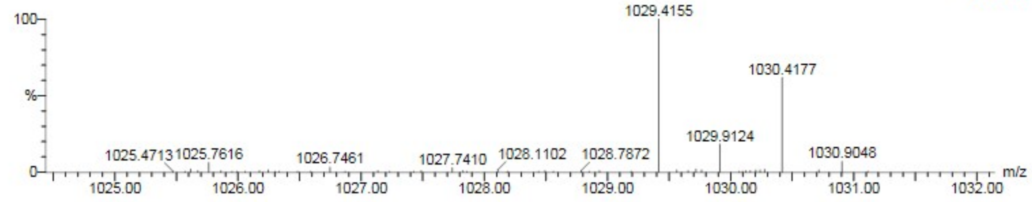

Minimum:				-1.5			
Maximum:		300.0	50.0	100.0			
Mass	Calc. Mass	mDa	PPM	DBE	i-FIT	i-FIT (Norm)	Formula
1029.4155	1029.4155	0.0	0.0	26.5	63.9	0.0	C56 H60 N4 O6 P F6

Figure S19. ESI-mass spectrum of compound **S4** ($[M-PF_6]^+$: 1029.4155)

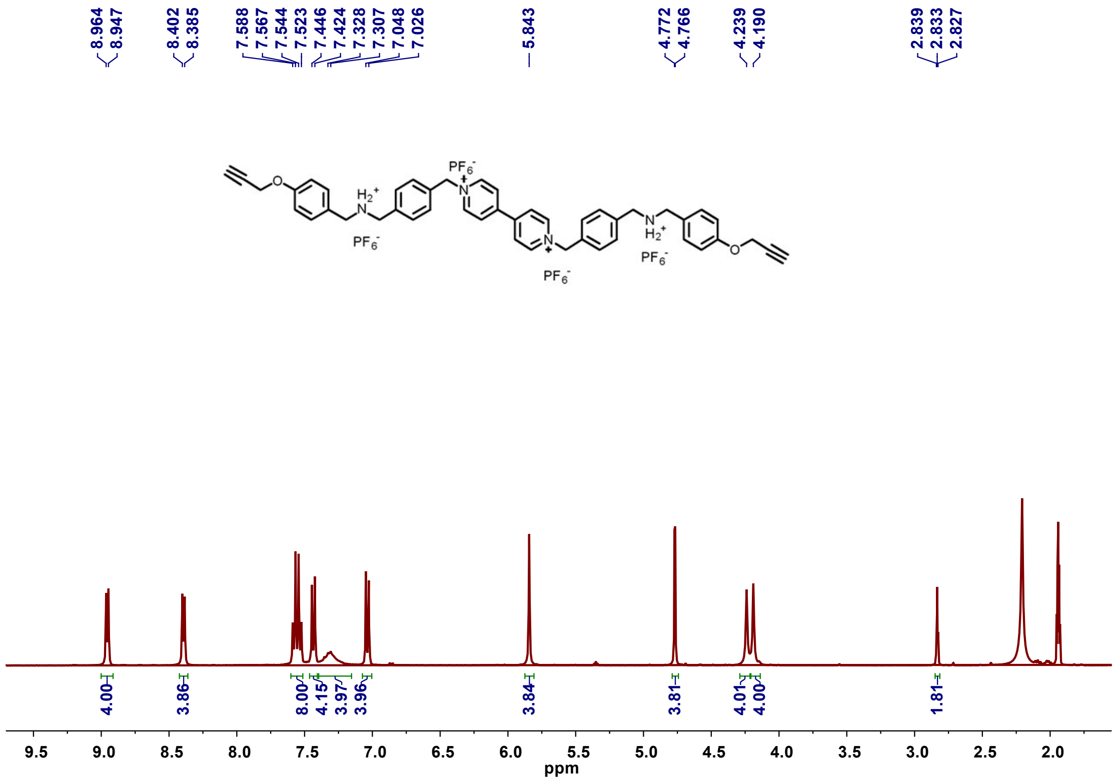

Figure S20. ^{1}H NMR spectrum of **1** (CD_3CN, 400MHz, 298K)

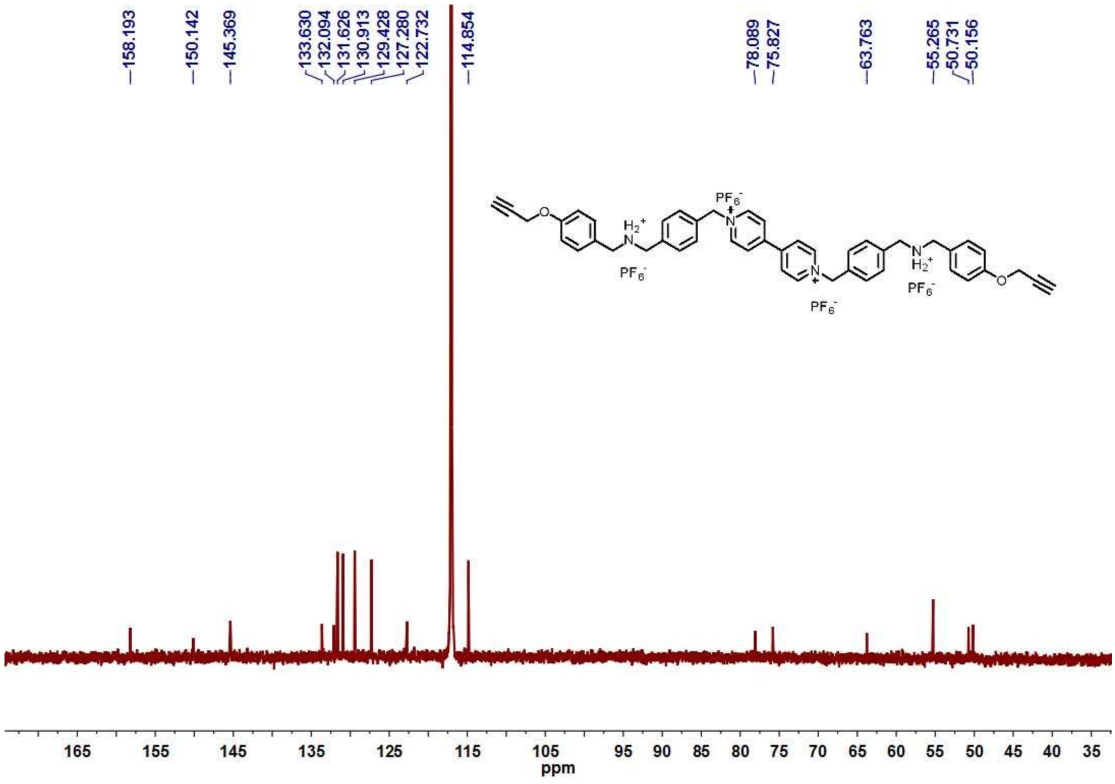

Figure S21. ^{13}C NMR spectrum of **1** (CD_3CN, 100MHz, 298K)

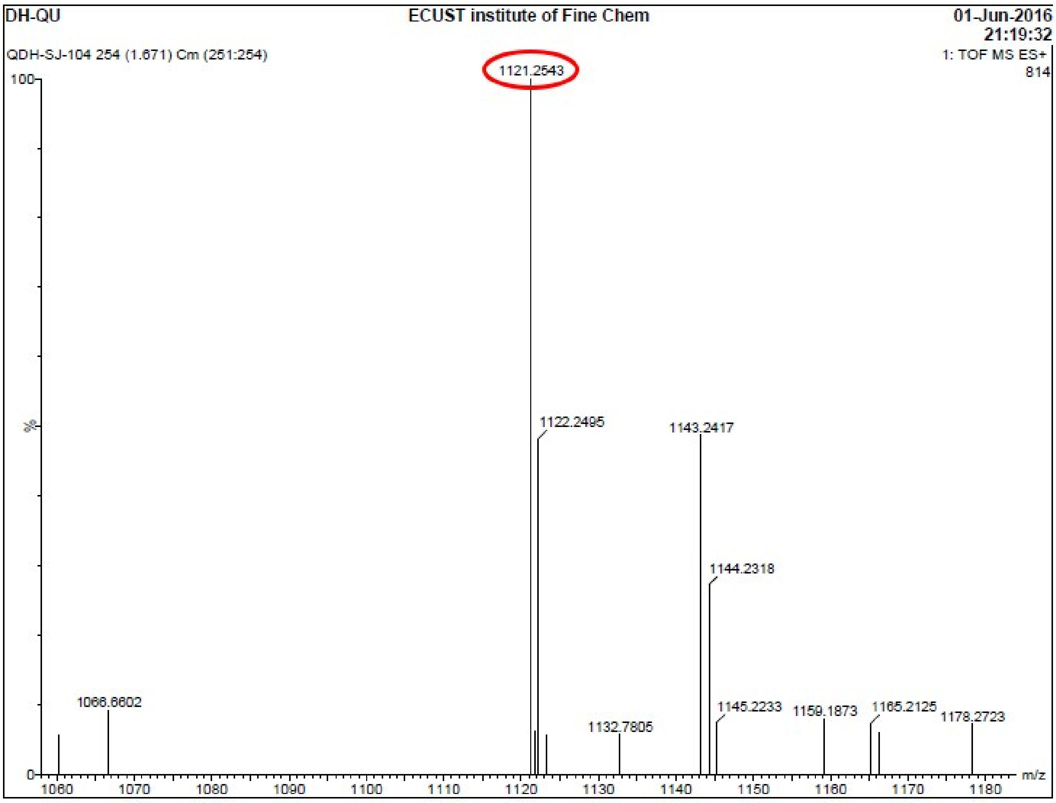

Figure S22. ESI-mass spectrum of compound **1** ($[M-PF_6]^+$: 1121.2543).

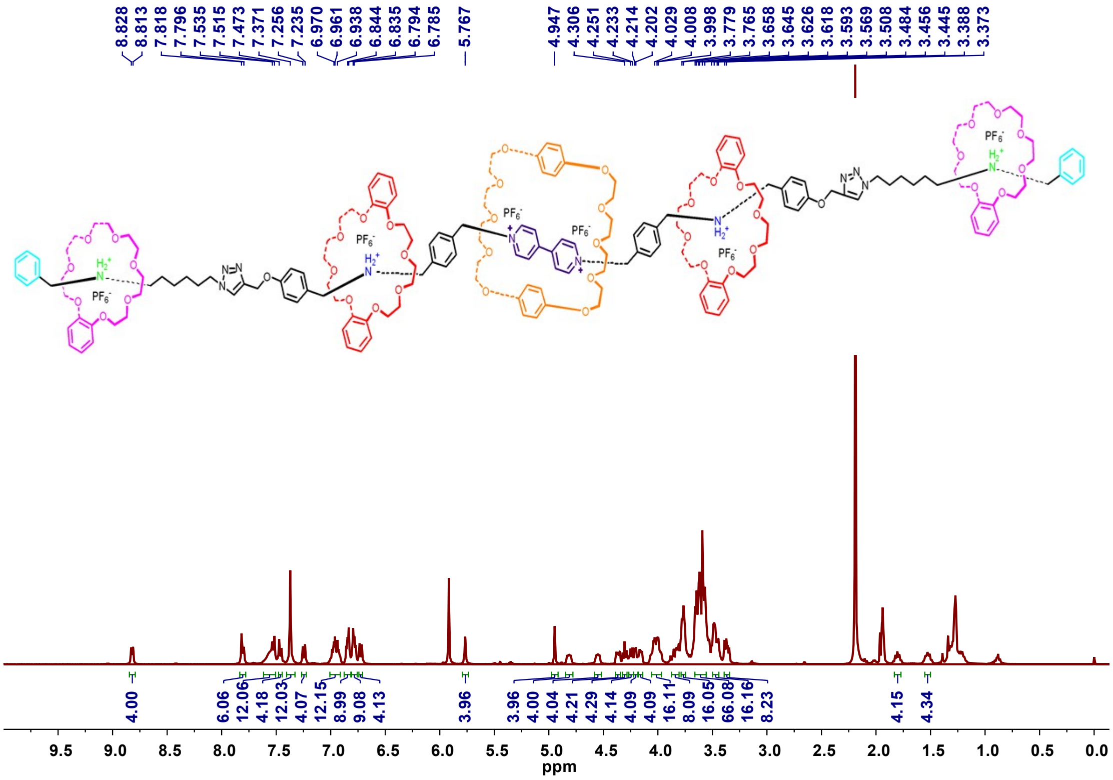

Figure S23. ^{1}HNMR spectrum of **8** (CD_3CN, 400MHz, 298K)

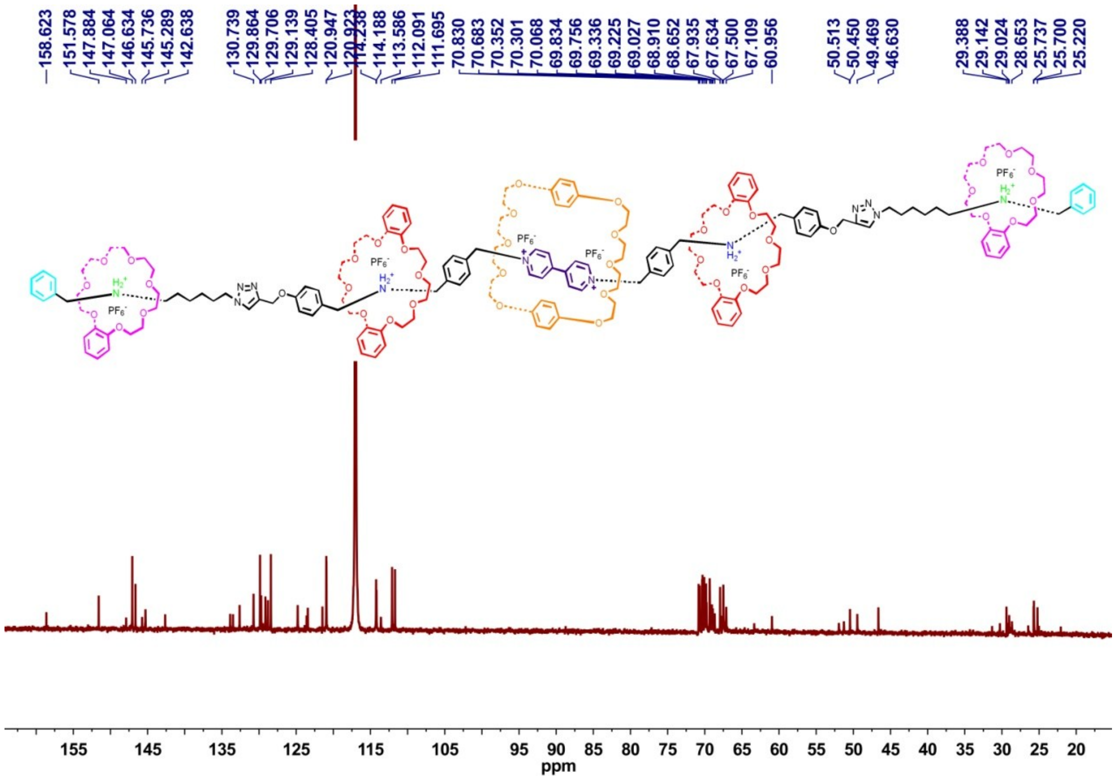

Figure S24. ^{13}C NMR spectrum of **8** (CD_3CN, 100MHz, 298K)

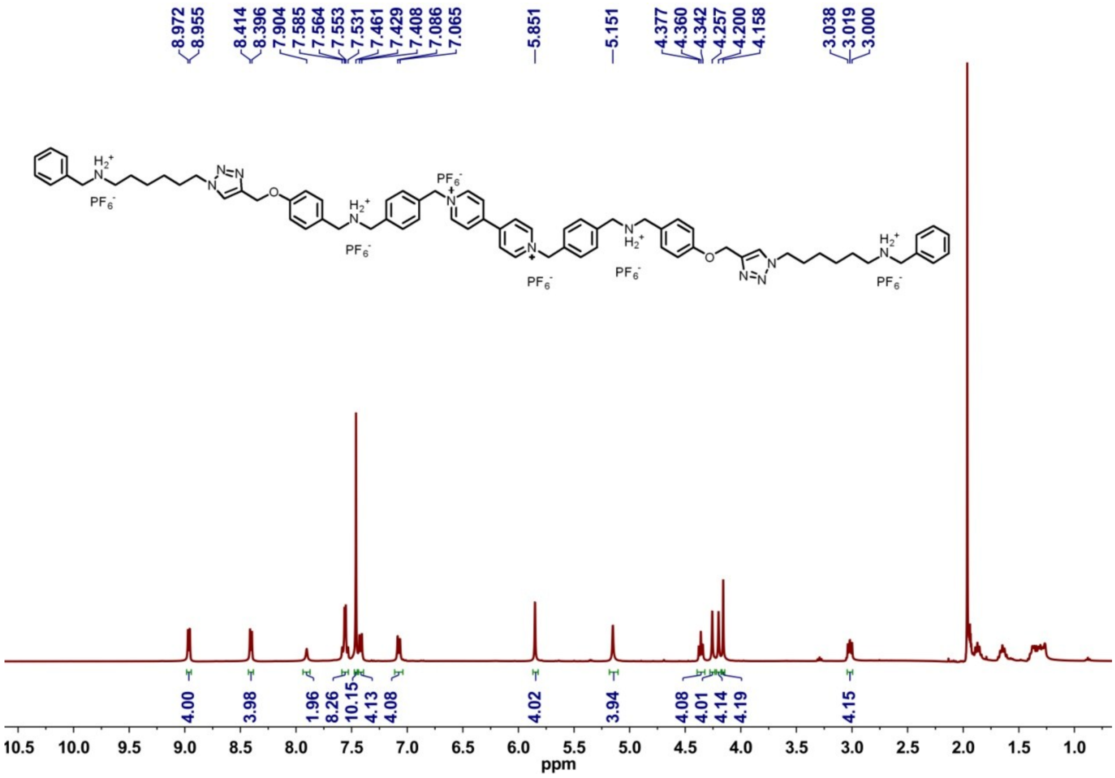

Figure S25. ^{1}H NMR spectrum of C**1** (CD_3CN, 400MHz, 298K)

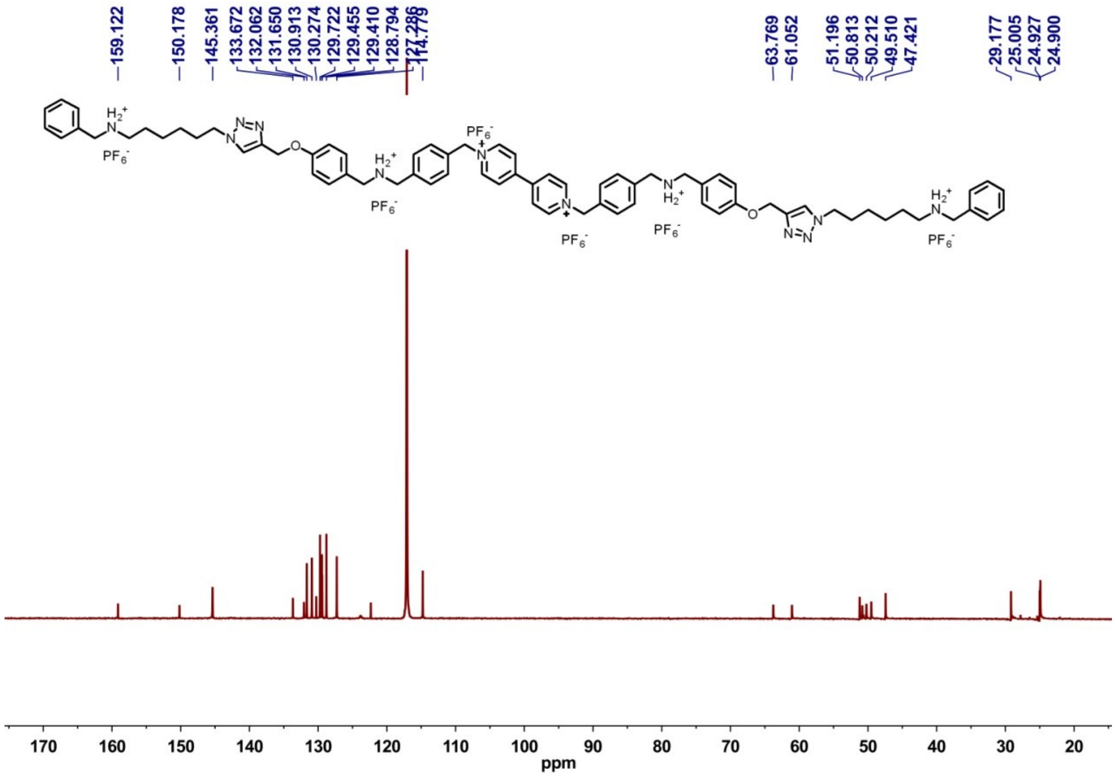

Figure S26. ^{13}C NMR spectrum of **C1** (CD_3CN, 100MHz, 298K)

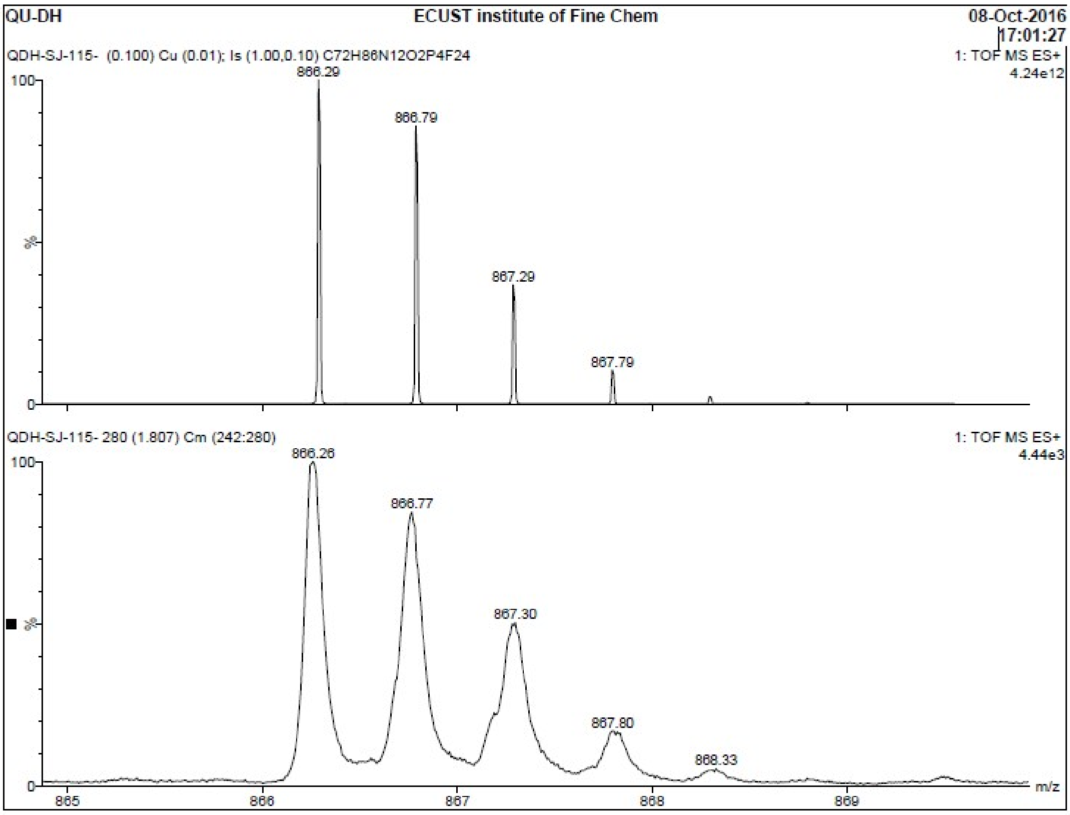

Figure S27. ESI-mass spectrum of compound **C1** ($[M{-}2PF_6]^{2+}$: 866.26).

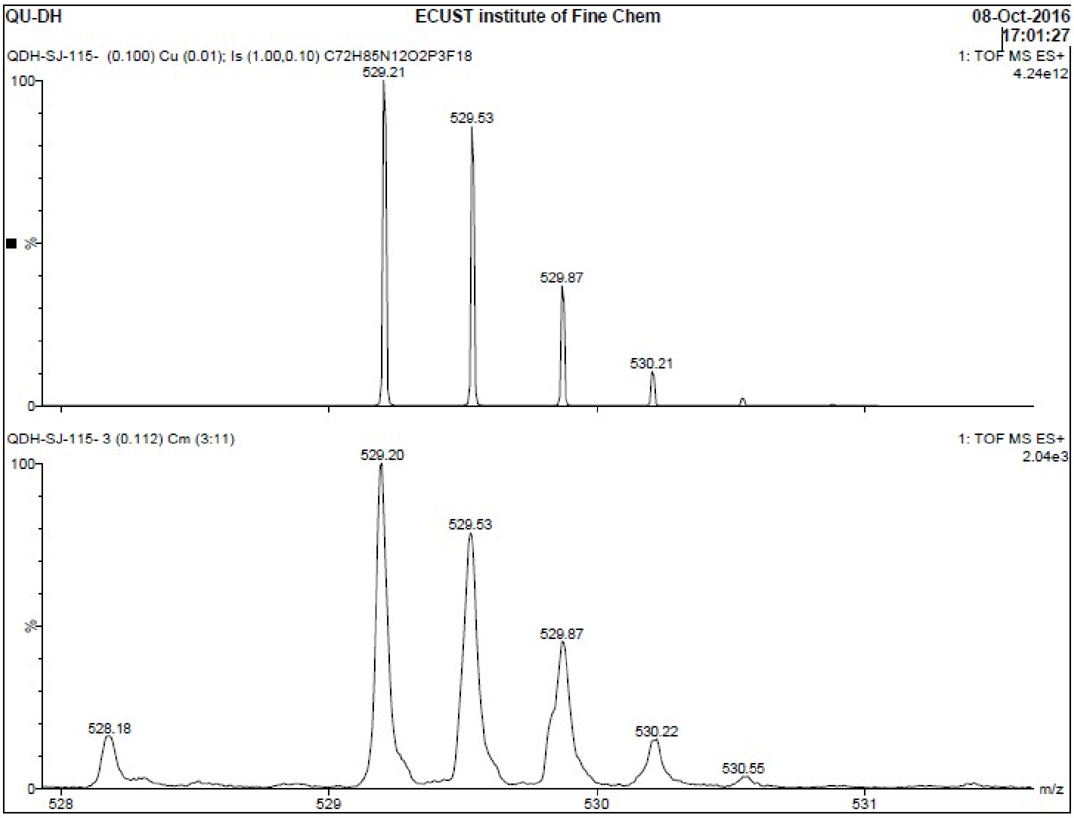

Figure S28. ESI-mass spectrum of compound **C1** ($[M{-}3PF_6]^{3+}$: 529.21).

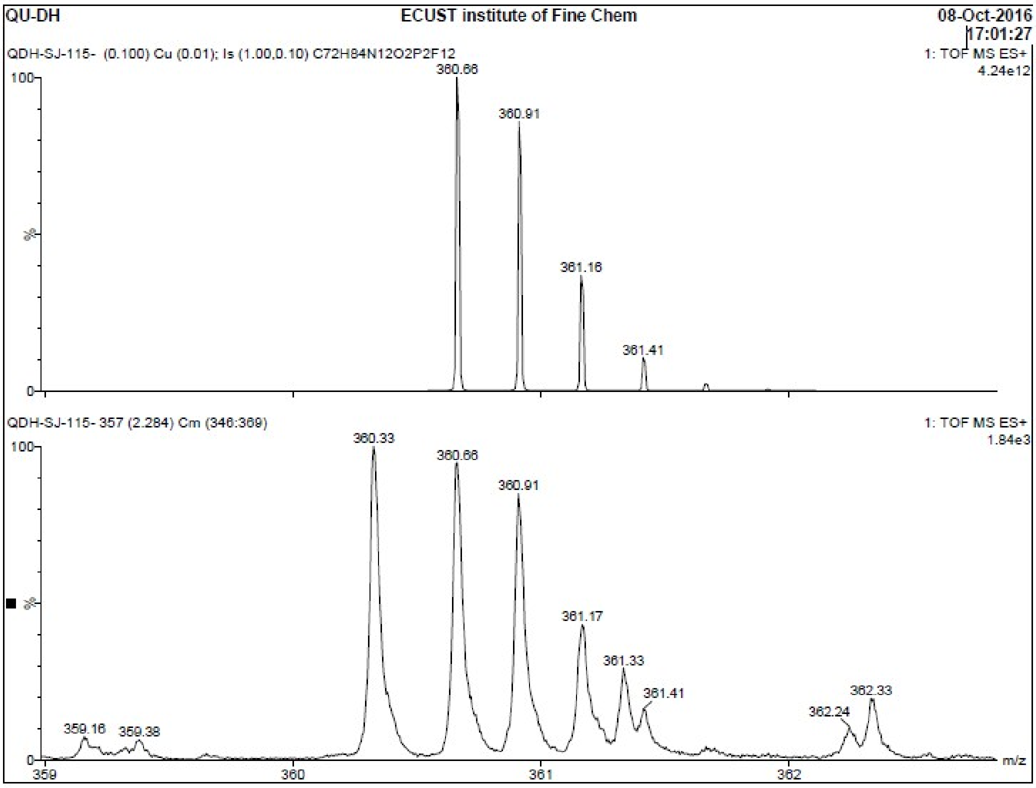

Figure S29. ESI-mass spectrum of compound **C1** ($[M{-}4PF_6]^{4+}$: 360.66).

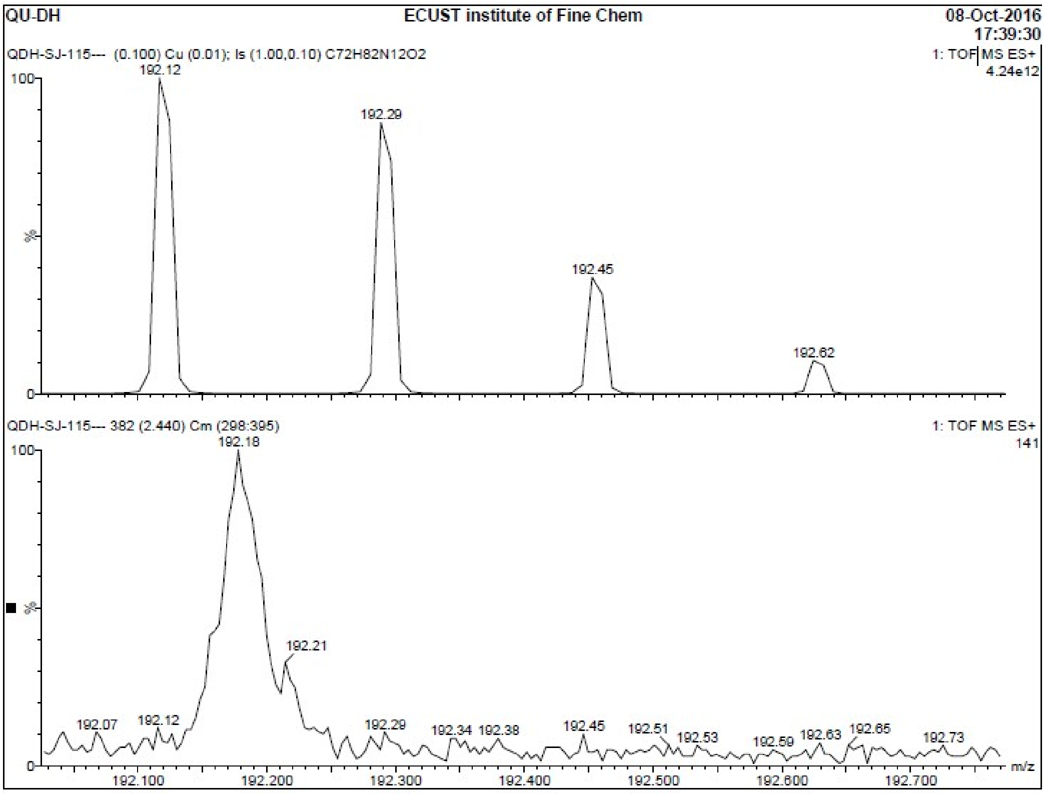

Figure S30. ESI-mass spectrum of compound **C1** ([M−6PF$_6$]$^{6+}$: 192.18).

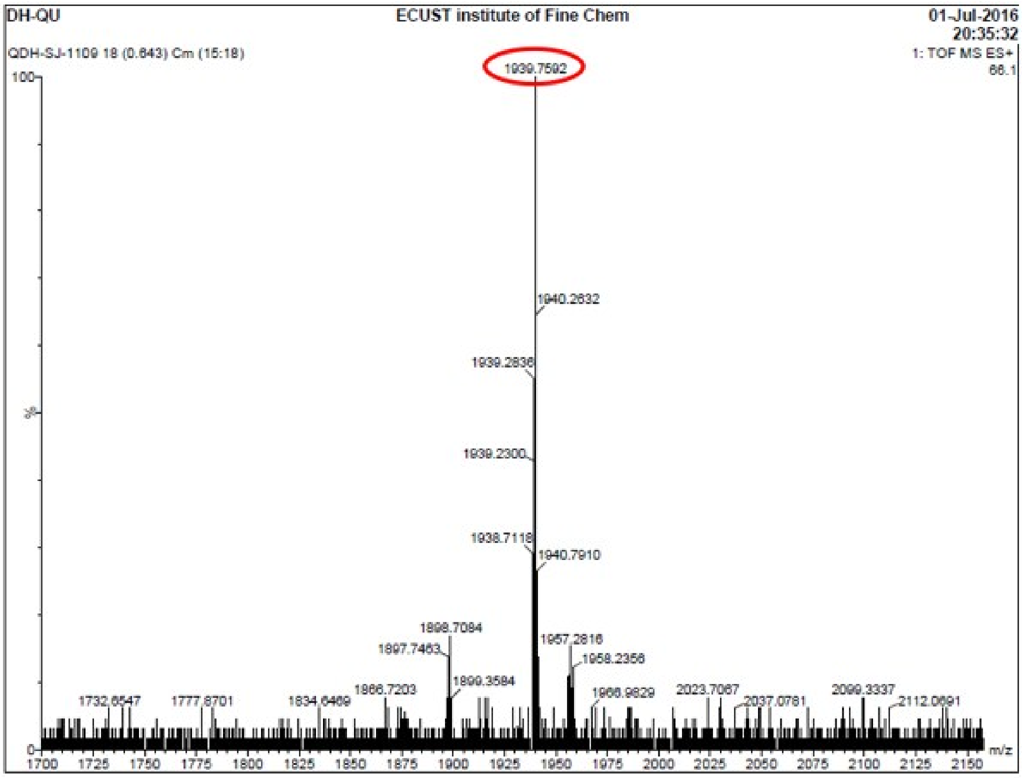

Figure S31. ESI-mass spectrum of hetero[6]rotaxane **8** ([M−2PF$_6$]$^{2+}$: 1939.7592).

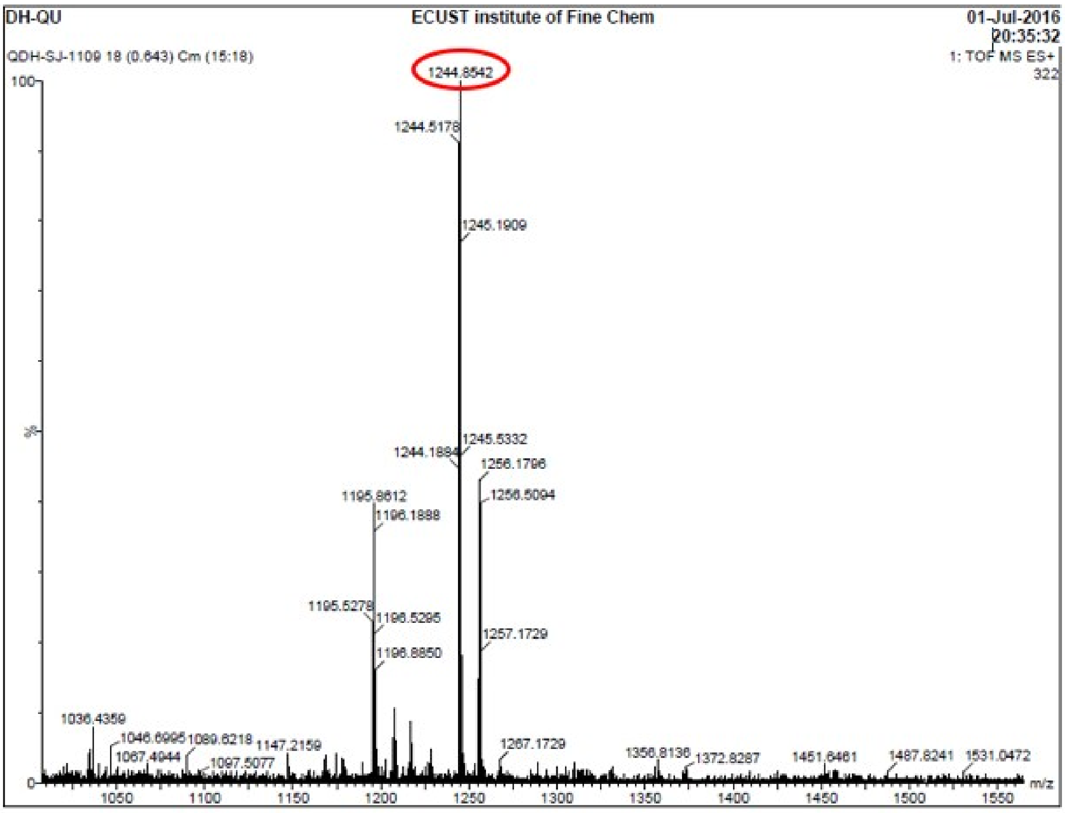

Figure S32. ESI-mass spectrum of hetero[6]rotaxane **8** ($[M{-}3PF_6]^{3+}$: 1244.8542).

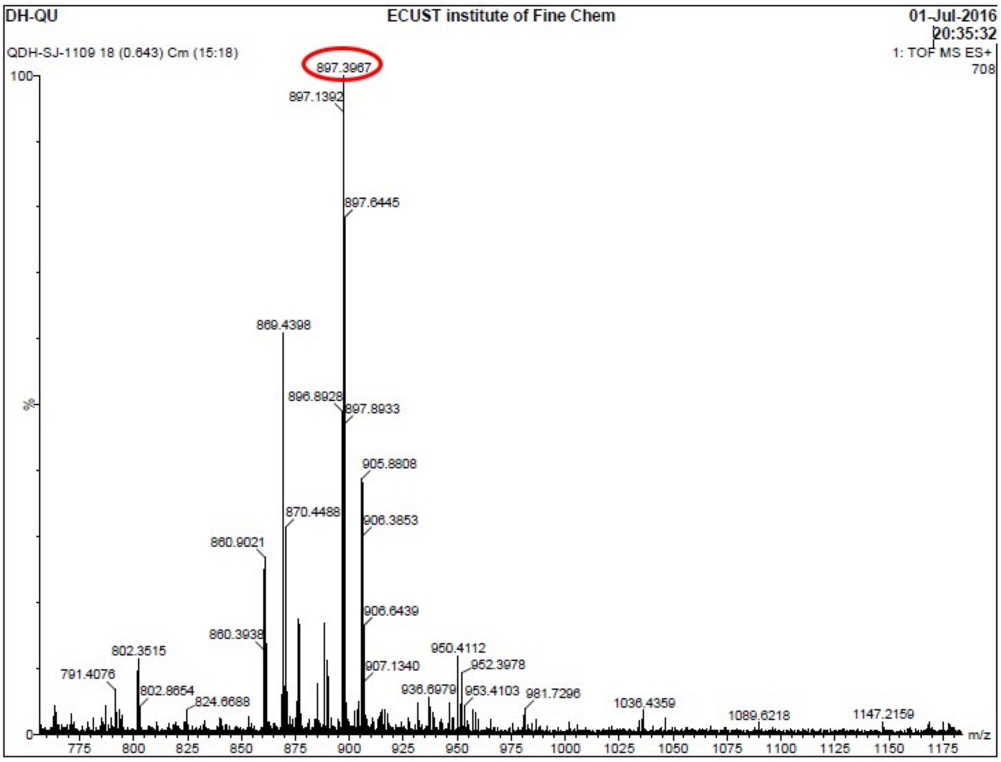

Figure S33. ESI-mass spectrum of hetero[6]rotaxane **8** ($[M{-}4PF_6]^{4+}$: 897.3967).

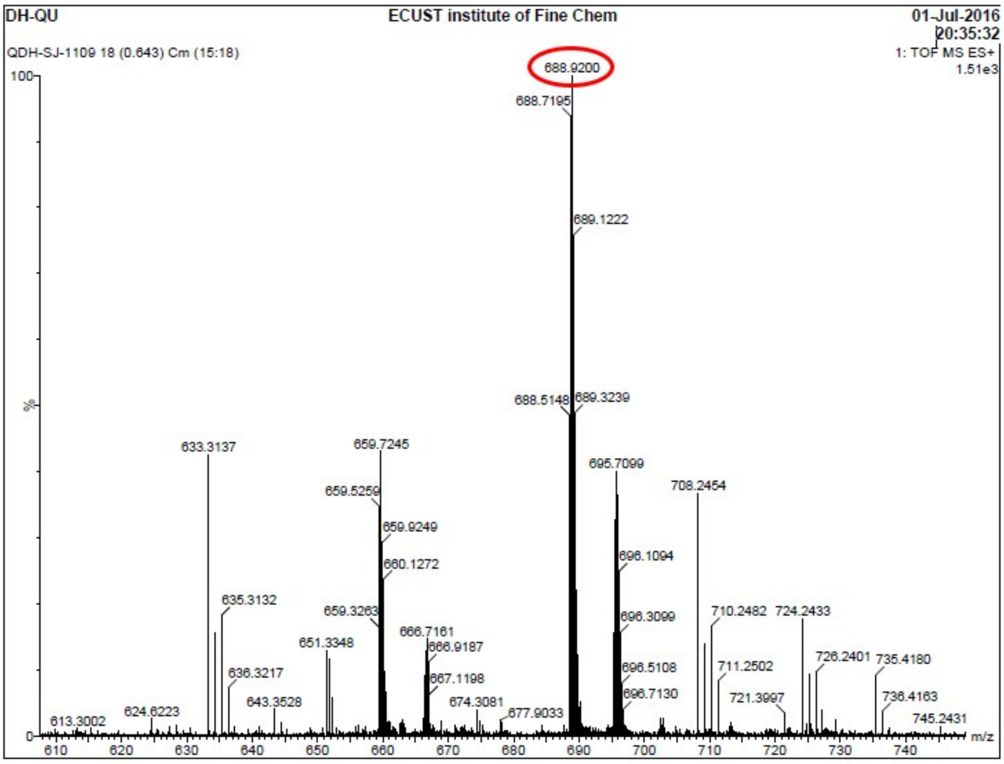

Figure S34. ESI-mass spectrum of hetero[6]rotaxane **8** ([M−5PF$_6$]$^{5+}$: 688.9200).

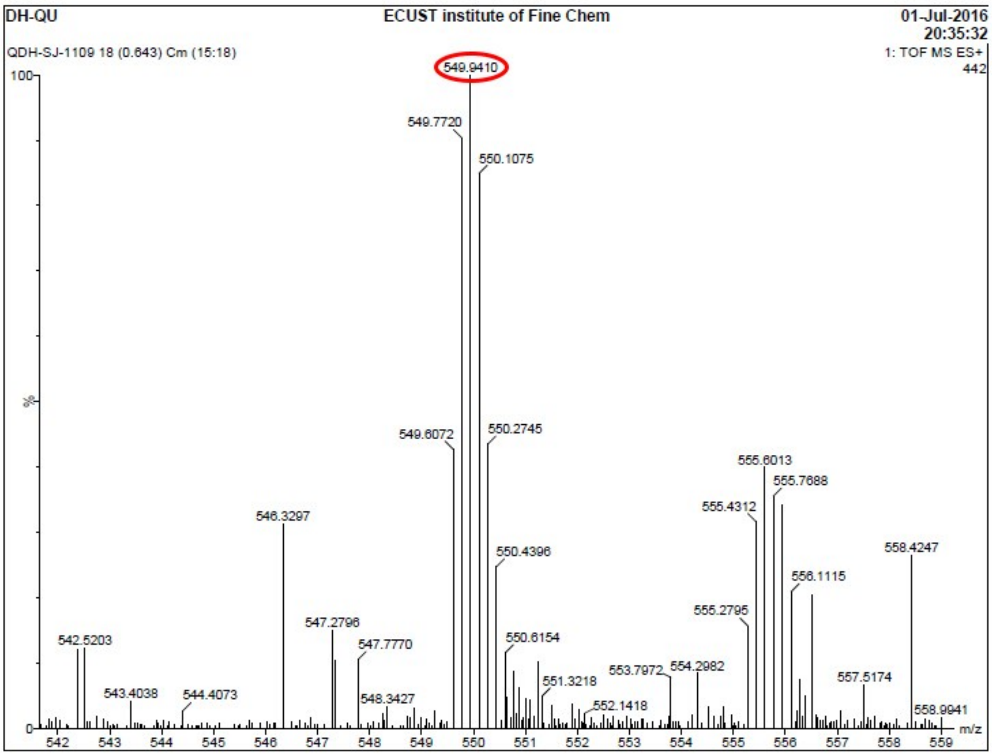

Figure S35. ESI-mass spectrum of hetero[6]rotaxane **8** ([M−6PF$_6$]$^{6+}$: 549.9410).

References:

(S1) Rauthu, S. R.; Shiao, T. C.; Andre, S.; Miller, M. C.; Madej, E.; Mayo, K. H.; Gabius, H. J.; Roy, R. *Chembiochem,* **2015,** *16,* 126.
(S2) Zhang, Z. J.; Zhang, H. Y.; Wang, H.; Liu, Y.; *Angew. Chem. Int. Ed.* **2011,** *123,* 11026.
(S3) Fu, X.; Zhang, Q.; Rao, S. J.; Qu, D. H.; Tian, H. *Chem. Sci.,* **2016,** *7,* 1696.
